# Distance and similarity measures for normal wiggly dual hesitant fuzzy sets and their application in medical diagnosis

**DOI:** 10.1038/s41598-022-16078-6

**Published:** 2022-08-12

**Authors:** Jawad Ali, Muhammad Naeem

**Affiliations:** 1 Institute of Numerical Sciences, Kohat University of Science and Technology, Kohat, KPK Pakistan; 2 Deanship of Joint First Year Umm Al-Qura University Makkah KSA, Mecca, Saudi Arabia

**Keywords:** Engineering, Mathematics and computing

## Abstract

The normal wiggly dual hesitant fuzzy set (NWDHFS) is a modern mathematical tool that can be used to express the deep ideas of membership and non-membership information hidden in the thought-level of decision-makers (DMs). To enhance and expand the applicability of NWDHFSs, this study originates several types of distance and similarity measures between two NWDHFSs. The present paper first revises the basic operational laws of normal wiggly dual hesitant fuzzy elements (NWDHFEs) and then generalizes the rule of length extension for normal wiggly dual hesitant fuzzy setting. Meanwhile, we introduce a variety of distance and similarity measures under the background of NWDHFSs. After that, a family of weighted distance and similarity measures based on NWDHFS is presented and analyzed for discrete and continuous cases. The stated measures are the extension of several existing measures and have the capability to handle uncertain and vague information with a wider range of information. DMs can select the most suitable alternative based on these measures by determining the gap between each alternative and the ideal one. Finally, a practical example concerning disease detection is addressed to demonstrate the applicability and merits of the developed theory and depict the differences between the presented distance and similarity measures.

## Introduction

Decision theory is an interdisciplinary approach that is used mainly in human activities. Since real decision-making problems are always created from a complicated context, the evaluation information is always vague. On that account, it is necessary to introduce some assistance tools in order to help DMs in making decisions. To do so, Zadeh^1^ coined the idea of fuzzy set (FS) as an extension of the classical notion of sets. Since its original definition, various extensions have been done for FSs, including type-2 fuzzy set^2^, intuitionistic fuzzy set (IFS)^3^, Pythagorean fuzzy set (PFS)^4,5^ and hesitant fuzzy set (HFS)^6^. The elements in IFS take into account membership as well as non-membership information. Owing to the consideration of non-membership information, the IFS is more efficient for practical implementations. Elaborate works on IFS have been conducted; see^7,8^ for detail. In many situations, due to limited knowledge or complexity of the world, DMs feel difficulty assigning only a single value during evaluation. HFS is quite helpful in avoiding such issues, which permits DMs to describe their description in terms of several possible values between 0 and 1. To date, lot of research work on aggregation operators^9–11^, distance and similarity measures^12–14^, correlation measures^15,16^ and decision making methods^17–19^ with hesitant fuzzy information have been done. Owing to its successful applications and some weaknesses, HFS has been explored in numerous formations such as picture hesitant fuzzy set^20^, necessary and possible hesitant fuzzy sets^21^, interval neutrosophic hesitant fuzzy set^22^, probabilistic hesitant fuzzy set^23^, expanded, and much more^24–26^. Recently, Ren et al.^27^ provided the theory of normal wiggly hesitant fuzzy set (NWHFS) in order to dig the deeper uncertain information in the hesitant fuzzy data. They studied the basic theory related to the proposed representation tool, including two preliminary aggregation operators, and applied them to the environmental pollution problem. Liu et al.^28^ developed normal wiggly hesitant fuzzy power Muirhead mean operators and utilized them to address decision-making problems. Further, Liu and Wang^29^ proposed the concept of normal wiggly hesitant fuzzy power generalized Maclaurin symmetric mean operators. Based on novel distance measures and operational laws of NWHFSs, Liu and Zhang^30^ put forward the correlation coefficient standard deviation (CCSD) method to determine the criteria weights objectively. They also connected the multi attributive border approximation area comparison (MABAC) method with prospect theory to cope with the MCDM problems under normal wiggly hesitant fuzzy setting. Yang et al.^31^ focused on solving the MCGDM issues with incomplete weight information by utilizing novel distance measures under the normal wiggly hesitant fuzzy environment. Besides, the authors of Ref.^32^ provided a decision-making framework for evaluating the best reinforcement of agro-waste while taking technical, environmental, and economic factors into account. In recent years, Ramya et al.^33^ employed normal wiggly hesitant fuzzy elimination and choice expressing reality (ELECTRE) method for the best versatile e-waste disposal technique selection. Due to the increasing complexity of the fundamental issues, NWHFS has been extended by many scholars, some of its extensions and their description is provided in Table 1.

**Table 1:** NWHFS extensions.

References	Title of the extension	Characteristic of the elements
^34^	Normal wiggly dual hesitant fuzzy set	Two sets of values from [0, 1] with possible membership and non-membership grades along deeper uncertain information such that the sum of the upper bounds of subintervals in membership and non-membership grades is less than or equal to 1
^35^	Normal wiggly pythagorean hesitant fuzzy set	Two sets of values from [0, 1] with possible membership and non-membership grades along deeper uncertain information such that the sum of values in membership and non-membership grades is less than or equal to 1
^36^	Normal wiggly probabilistic hesitant fuzzy set	A set of values from [0, 1] with possible probabilistic membership grades along deeper uncertain information
^37^	Normal wiggly interval-valued hesitant pythagorean fuzzy set	Two sets of subintervals from [0, 1] with possible membership and non-membership grades along deeper uncertain information such that the square sum of the upper bounds of subintervals in membership and non-membership grades is less than or equal to 1

Sometimes the accurate membership grades of evaluation information are cumbersome to be determined, which is the major shortcoming of HFS and its extensions. To tackle this issue, Zhu et al.^38^ explored the definition and related theory of dual hesitant fuzzy set (DHFS). Until now, several researchers have done a lot of work to further investigate the theory of DHFS, for instance, the distance and similarity measures^39–41^, the correlation measures^42,43^, the entropy measures^44–46^, and so on. Hsu et al.^47^ reviewed and examined the applications of DHFS in a probabilistic manner. Alcantud et al.^48^ put forward the superior concept of dual extended hesitant fuzzy set and discussed a comparison law for the prioritization of elements described in the proposed tool. Karaaslan and Özlü^49^ introduced dual type-2 hesitant fuzzy set and detailed its correlation coefficient formulas. Inspired by the idea of NWHFS, Narayanamoorthy et al.^34^ presented the notion of NWDHFS and discussed its application to site selection. NWDHFS takes DHFS as the original information, from which it digs the potential uncertain information of the DMs in order to get the complete evaluation information.

Distance and similarity measures are key concepts in decision-making, especially machine learning, pattern recognition, image processing, medical diagnosis, scheme selection, etc. So far, numerous researches have been conducted on this topic^12–14^. Originally, Wang^50^ initiated the concept of FSs’ similarity measure with a mathematical formula. The most popular and widely used distance measures for two FSs $A_1$ and $A_2$ on $\textit{X}$ are the following^51–53^:

- Hamming distance: $$d_h\left( A_1, A_2\right) =\sum _{j=1}^{n}\left| \mu _{A_1}(\textit{x}_{j})-\mu _{A_2}(\textit{x}_{j})\right| ;$$
- Euclidean distance: $$d_e\left( A_1, A_2\right) =\left( \sum _{j=1}^{n}\left| \mu _{A_1}(\textit{x}_{\jmath })-\mu _{A_2}(\textit{x}_{j})\right| ^{2}\right) ^{1/2};$$
- Hausdorff metric: $$\max \left| \mu _{A_1}(\textit{x}_{j})-\mu _{A_2}(\textit{x}_{j})\right| ,$$

where $\mu _{A_1}(\textit{x}_j)$ and $\mu _{A_2}(\textit{x}_j)$ are the membership grades of $A_1$ and $A_2$, respectively, meet the condition that $0\le \mu _{A_1}(\textit{x}_j), \mu _{A_2}(\textit{x}_j)\le 1$, for $\textit{x}_j \in \textit{X}, j=1,2,...,n.$

Later, numerous scholars paid attention to this topic and expanded further. Several distance and similarity measures have been developed for FS, IFS, HFS, DHFS, etc. Szmidt and Kacprzyk^54^ proposed a novel distance between two IFS. Xia and Xu^55^ studied the distance and similarity measure based on HFS and studied their application in decision-making problems. Peng et al.^56^ gave various distance, similarity, entropy, and inclusion measures for Pythagorean fuzzy set and their relationships between them. Khan and his coworkers^57^ studied set-theoretic distance and similarity measures for spherical fuzzy sets (SFS) and showed their application in selecting mega projects problem. Liu et al.^58^ illustrated some distance measures for DHFS based on connection numbers and discussed several identities and relationships between them. Zhang et al.^59^ investigated some improved distance measures for HFSs and DHFSs to avoid the issue of extension process in the previous distance measures. They also provide various entropy measures for DHFSs, which describe the fuzziness of DHFSs. Recently, Wang et al.^60^ proposed novel distance measures in terms of mean and variance for dual hesitant fuzzy setting and utilized them in practical problems.

Keeping in mind the importance of distance and similarity measures and application in decision-making, medical diagnosis, and pattern recognition, numerous authors, have done much work on this topic until now. However, there is no research on distance and similarity measures based on NWDHFS. To study the MCGDM techniques viz. TOPSIS, GLDS, TODIM, VIKOR, and ELECTRE for NWHFSs, there is an urgent need to design normal wiggly hesitant fuzzy distance and similarity measures. Therefore, this study aims to generalize traditional distance and similarity measures and their weighted forms for both discrete and continuous cases with respect to normal wiggly dual hesitant fuzzy context.

The rest of the paper is organized in the following manner: section “Preliminaries” recalls some preliminary knowledge related to DHFSs and NWDHFSs, including the revised operational laws of NWDHFEs. Section “Distance and similarity measures between two NWDHFSs” first generalizes the rule of length extension and then gives the definitions of distance and similarity measures for NWDHFSs, based on which various distance and similarity measures for two NWDHFSs are developed. In section “Weighted distance and similarity measures between two NWDHFSs”, we propose a variety of weighted distance and similarity measures under the normal wiggly dual hesitant fuzzy environment for both discrete and continuous cases, respectively. Section “Application of the proposed distance (similarity) measures” illustrates a real-world example to show the practicality and performance of our measures. Lastly, section “Concluding remarks and suggestions” concludes the paper with some remarks and presents future challenges.

## Preliminaries

This section presents some basic concepts related to DHFS and NWDHFS, including the revised operational laws of NWDHFSs.

### Definition 1

^38^ Let $\textit{X}$ be a reference set, then a DHFS $D_s$ on $\textit{X}$ is given by:

1 $$\begin{aligned} D_s=\left\{ \left\langle \textit{x},h_s(\textit{x}), g_s(\textit{x}) \right\rangle | \textit{x} \in X \right\} , \end{aligned}$$

where $h_s(\textit{x})$ and $g_s(\textit{x})$ are two sets of numbers from [0, 1], representing the possible membership degrees and non-membership degrees of the element $\textit{x}\in X$ to the set $D_s$ respectively, with the condition that $0 \le \max \left( h_s(\textit{x}) \right) + \max \left( g_s(\textit{x}) \right) \le 1$ . For convince, the element $d_s(\textit{x})=\left( h_s(\textit{x}), g_s(\textit{x}) \right)$ is called the dual hesitant fuzzy element (DHFE), which can be simply marked as $d_s=\left( h_s ,g_s \right)$.

### Definition 2

^61^ Let $d_s=\left( h_s ,g_s \right)$; $h_s=\left\{ \alpha _1, \alpha _2,...,\alpha _{\#h_s}\right\}$ and $g_s=\left\{ \beta _1, \beta _2,...,\beta _{\#g_s}\right\}$ be a DHFE. Then, the mean and the standard deviation of $h_s$ and $g_s$ are given as:

2 $$\begin{aligned}&\overline{h_s}=\sum _{\imath =1}^{\# h_s}\alpha _{\imath }/\# h_s , \end{aligned}$$

3 $$\begin{aligned}&\overline{g_s}=\sum _{\imath =1}^{\# g_s}\beta _{\imath }/\# g_s , \end{aligned}$$

and

4 $$\begin{aligned} & \sigma _{h_s}=\sqrt{\sum _{\imath =1}^{\# h_s}\left( \alpha _{\imath }-\overline{h_s}\right) ^{2}/\# h_s}, \end{aligned}$$

5 $$\begin{aligned} & \sigma _{g_s}=\sqrt{\sum _{\imath =1}^{\# g_s}\left( \beta _{\imath }-\overline{g_s}\right) ^{2}/\# g_s}, \end{aligned}$$

respectively. The functions ${\widetilde{f}}:h_s \longrightarrow \left[ 0,\sigma _{h_s}\right]$ and ${\widetilde{f}}:g_s \longrightarrow \left[ 0,\sigma _{g_s}\right]$ satisfying ${\widetilde{f}}\left( \alpha _{\imath } \right) =\sigma _{h_s}e^-\frac{\left( \alpha _{\imath }-\overline{h_s}\right) ^2}{2\sigma ^2_{h_s}}$ and ${\widetilde{f}}\left( \beta _{\imath } \right) =\sigma _{g_s}e^-\frac{\left( \beta _{\imath }-\overline{g_s}\right) ^2}{2\sigma ^2_{g_s}}$ are nominated as the normal wiggly range (NWR) of $\alpha _{\imath }$ and $\beta _{\imath }$, respectively.

### Definition 3

^61^ Let $d_s=\left( h_s ,g_s \right)$; $h_s=\left\{ \alpha _1, \alpha _2,...,\alpha _{\#h_s}\right\}$ and $g_s=\left\{ \beta _1, \beta _2,...,\beta _{\#g_s}\right\}$ be a DHFE. Further, let $\widetilde{d_s} =\left( {\widetilde{h_{s}}} ,{\widetilde{g_{s}}} \right)$; ${\widetilde{h_{s}}}=\left\{ \widetilde{\alpha }=\alpha _{\imath }/\sum _{\imath =1}^{\#h_s } \alpha _{\imath } | \alpha _{\imath } \in h_s\right\}$ and ${\widetilde{g_{s}}}=\left\{ \widetilde{\beta }=\beta _{\imath }/\sum _{\imath =1}^{\#g_s } \beta _{\imath } | \beta _{\imath } \in g_s\right\}$ be a normalized DHFE. Then, the real preference degrees (rpd) of ${\widetilde{h_{s}}}$ and ${\widetilde{g_{s}}}$ are given by

$$\begin{aligned}&\text {rpd}\left( {\widetilde{h_{s}}} \right) ={\left\{ \begin{array}{ll} \sum _{\imath =1}^{\#{\widetilde{h_{s}}}}\widetilde{\alpha _{\imath }}\left( \frac{\#{\widetilde{h_{s}}} -\imath }{{\widetilde{h_{s}}} -1} \right) , &{} \text{ if } \, \overline{h_s}< 0.5\\ 1- \sum _{\imath =1}^{\#{\widetilde{h_{s}}}}\widetilde{\alpha _{\imath }}\left( \frac{\#{\widetilde{h_{s}}} -\imath }{{\widetilde{h_{s}}} -1} \right) , &{} \text{ if } \, \overline{h_s}> 0.5 \\ 0.5, &{} \text{ if } \, \overline{h_s}= 0.5. \end{array}\right. } \\&\text {rpd}\left( {\widetilde{g_{s}}} \right) ={\left\{ \begin{array}{ll} \sum _{\imath =1}^{\#{\widetilde{g_{s}}}}\widetilde{\beta _{\imath }}\left( \frac{\#{\widetilde{g_{s}}} -\imath }{{\widetilde{g_{s}}} -1} \right) , &{} \text{ if } \, \overline{g_s} < 0.5\\ 1- \sum _{\imath =1}^{\#{\widetilde{g_{s}}}}\widetilde{\beta _{\imath }}\left( \frac{\#{\widetilde{g_{s}}} -\imath }{{\widetilde{g_{s}}} -1} \right) , &{} \text{ if } \, \overline{g_s} > 0.5 \\ 0.5, &{} \text{ if } \,\overline{g_s}= 0.5. \end{array}\right. } \end{aligned}$$

which are measured based on the orness measure, originally proposed by Yager^62^.

### Definition 4

^34^ Let $H_s=\left\{ \textit{x}, h_s(\textit{x}) | \textit{x} \in \textit{X}\right\}$ be a DHFS on the reference set $\textit{X}$. Then, the NWDHFS on $\textit{X }$ can be described as:

6 $$\begin{aligned} N_s=\left\{ \left\langle \textit{x}, h_s(\textit{x}), g_s(\textit{x}), \varphi \left( h_s(\textit{x}) \right) , \varphi \left( g_s(\textit{x}) \right) \right\rangle | \textit{x} \in \textit{X } \right\} , \end{aligned}$$

where $$\varphi \left( h_s(\textit{x}) \right) =\left\{ {\mathop{{\alpha}}\limits^{\frown}}_{1}, {\mathop{{\alpha}}\limits^{\frown}}_{2},...,{\mathop{{\alpha}}\limits^{\frown}}_{\#h_s(\textit{x})} \right\} $$, $${\mathop{{\alpha}}\limits^{\frown}}_{\imath }=\left\{ a_{\imath }^{L},a_{\imath }^{M},a_{\imath }^{U}\right\} =\left\{ \max \left( \alpha _{\imath } -{\widetilde{f}} \left( \alpha _{\imath }\right) ,0 \right) , \right. $$

$\left. \left( 2\text {rpd}\left( {\widetilde{h_{s}}} (\textit{x})\right) -1\right) {\widetilde{f}}\left( \alpha _{\imath }\right) +\alpha _{\imath }, \min \left( \alpha _{\imath }+{\widetilde{f}}\left( \alpha _{\imath } \right) ,1 \right) \right\} ,$ $\alpha _{\imath }$ is one of the value of $h_s(\textit{x})$. Similarly, $\varphi \left( g_s(\textit{x}) \right) =\left\{ {\mathop{{\beta}}\limits^{\frown}}_{1} , {\mathop{{\beta}}\limits^{\frown}}_{2},...,{\mathop{{\beta}}\limits^{\frown}}_{\#h_s(\textit{x})} \right\}$, ${\mathop{{\beta}}\limits^{\frown}}_{\imath }=\left\{ b_{\imath }^{L},b_{\imath }^{M},b_{\imath }^{U}\right\} =\left\{ \max \left( \beta _{\imath } -{\widetilde{f}} \left( \beta _{\imath }\right) ,0 \right) , \right.$

$\left. \left( 2\text {rpd}\left( {\widetilde{g_{s}}} (\textit{x})\right) -1\right) {\widetilde{f}}\left( \alpha _{\imath }\right) +\alpha _{\imath }, \min \left( \beta _{\imath }+{\widetilde{f}}\left( \beta _{\imath } \right) ,1 \right) \right\} ,$ $\beta _{\imath }$ is one of the value of $g_s(\textit{x})$.

Also, ${\widetilde{f}} \left( \alpha _{\imath }\right)$ and ${\widetilde{f}} \left( \beta _{\imath }\right)$ are the wiggly parameters of $\alpha _{\imath }$, $\beta _{\imath }$ and rpd$\left( {\widetilde{h_{s}}} (\textit{x})\right)$ and rpd$\left( {\widetilde{g_{s}}} (\textit{x})\right)$ are the real preference degree of $h_s(\textit{x}), g_s(\textit{x}).$ Moreover, $\varphi \left( h_s(\textit{x}) \right)$ and $\varphi \left( g_s(\textit{x}) \right)$ are called normal wiggly elements (NWEs) and the pairs $\left\langle h_s(\textit{x}) , \varphi \left( h_s(\textit{x}) \right) \right\rangle$ $\left\langle g_s(\textit{x}) , \varphi \left( g_s(\textit{x}) \right) \right\rangle$ are called normal wiggly dual hesitant fuzzy elements (NWDHFEs), simply marked as $\left\langle h_s , g_s, \varphi \left( h_s \right) ,\varphi \left( h_s \right) \right\rangle$.

It should be pointed out that the original operational laws of NWDHFEs presented by^34^, is missing and insensible. Anyhow, the refined form of that operational laws is provided below.

### Definition 5

Let $\left\langle h^1_s , g^1_s , \varphi \left( h^1_s \right) , \varphi \left( g^1_s \right) \right\rangle$ and $\left\langle h^2_s , g^2_s , \varphi \left( h^2_s \right) , \varphi \left( g^2_s \right) \right\rangle$ be any two NWDHFEs, and $\lambda > 0$, then

1. $$\begin{aligned}&\left\langle h^1_s , g^1_s , \varphi \left( h^1_s \right) , \varphi \left( g^1_s \right) \right\rangle \oplus \left\langle h^2_s , g^2_s , \varphi \left( h^2_s \right) , \varphi \left( g^2_s \right) \right\rangle \\&\quad =\left\langle \begin{array}{l} \bigcup \limits _{\alpha _1 \in h^1_s, \alpha _2 \in h^2_s} \alpha _1 +\alpha _2 - \alpha _1 \alpha _2 ,\bigcup \limits _{\beta _1 \in g^1_s, \beta _2 \in g^2_s} \beta _1 \beta _2 , \\ \bigcup \limits _{{\mathop{{\alpha}}\limits^{\frown}}_{1} \in \varphi \left( h^1_s \right) , {\mathop{{\alpha}}\limits^{\frown}}_{2} \in \varphi \left( h^2_s \right) } {\mathop{{\alpha}}\limits^{\frown}}_{1} \oplus {\mathop{{\alpha}}\limits^{\frown}}_{2} \bigcup \limits _{{\mathop{{\beta}}\limits^{\frown}}_{1} \in \varphi \left( g^1_s \right) , {\mathop{{\beta}}\limits^{\frown}}_{2} \in \varphi \left( g^2_s \right) } {\mathop{{\beta}}\limits^{\frown}}_{1} \oplus {\mathop{{\beta}}\limits^{\frown}}_{2}\end{array}\right\rangle ; \end{aligned}$$
2. $$\begin{aligned}&\left\langle h^1_s , g^1_s , \varphi \left( h^1_s \right) , \varphi \left( g^1_s \right) \right\rangle \otimes \left\langle h^2_s , g^2_s , \varphi \left( h^2_s \right) , \varphi \left( g^2_s \right) \right\rangle \\&\quad =\left\langle \begin{array}{l} \bigcup \limits _{\alpha _1 \in h^1_s, \alpha _2 \in h^2_s} \alpha _1 \alpha _2 ,\bigcup _{\beta _1 \in g^1_s, \beta _2 \in g^2_s} \beta _1+\beta _2- \beta _1 \beta _2 , \\ \bigcup \limits _{{\mathop{{\alpha}}\limits^{\frown}}_{1} \in \varphi \left( h^1_s \right) , {\mathop{{\alpha}}\limits^{\frown}}_{2} \in \varphi \left( h^2_s \right) } {\mathop{{\alpha}}\limits^{\frown}}_{1} \otimes {\mathop{{\alpha}}\limits^{\frown}}_{2} \bigcup \limits _{{\mathop{{\beta}}\limits^{\frown}}_{1} \in \varphi \left( g^1_s \right) , {\mathop{{\beta}}\limits^{\frown}}_{2} \in \varphi \left( g^2_s \right) } {\mathop{{\beta}}\limits^{\frown}}_{1} \otimes {\mathop{{\beta}}\limits^{\frown}}_{2} \end{array}\right\rangle ; \end{aligned}$$
3. $$\begin{aligned} \left\langle h^1_s , g^1_s, \varphi \left( h^1_s \right) ,\varphi \left( g^1_s \right) \right\rangle ^{\lambda }=\left\langle \bigcup _{\alpha _1 \in h^1_s}\alpha ^{\lambda }_1, \bigcup _{\beta _1 \in g^1_s} 1- \left( 1- \beta _1 \right) ^{\lambda }, \bigcup _{{\mathop{{\alpha}}\limits^{\frown}}_{1} \in \varphi \left( h^1_s \right) }{{\mathop{{\alpha}}\limits^{\frown}}}^{\lambda }_1,\bigcup _{{\mathop{{\beta}}\limits^{\frown}}_{1} \in \varphi \left( g^1_s \right) }{{\mathop{{\beta}}\limits^{\frown}}}^{\lambda }_1 \right\rangle ; \end{aligned}$$
4. $$\begin{aligned} \lambda \left\langle h^1_s , \varphi \left( h^1_s \right) \right\rangle =\left\langle \bigcup _{\alpha _1 \in h^1_s}1- \left( 1-\alpha _1\right) ^{\lambda }, \bigcup _{\beta _1 \in g^1_s} \beta _1^{\lambda }, \bigcup _{{\mathop{{\alpha}}\limits^{\frown}}_{1} \in \varphi \left( h^1_s \right) }{\lambda }{\mathop{{\alpha}}\limits^{\frown}}_1, \bigcup _{{\mathop{{\beta}}\limits^{\frown}}_{1} \in \varphi \left( g^1_s \right) }{\lambda }{\mathop{{\beta}}\limits^{\frown}}_1 \right\rangle . \end{aligned}$$

With regards to distinguish the NWDHFEs, Narayanamoorthy et al.^34^ provided the following score function:

### Definition 6

Let $\left\langle h_s , g_s, \varphi \left( h_s \right) ,\varphi \left( g_s \right) \right\rangle$ be a NWDHFE. Then, the score function of $\left\langle h_s , g_s, \varphi \left( h_s \right) ,\varphi \left( g_s \right) \right\rangle$ is expressed as

7 $$\begin{aligned} S_{N_s} \left( \left\langle h_s, g_s, \varphi \left( h_s \right) ,\varphi \left( g_s \right) \right\rangle \right) =\left[ \delta \left( \overline{h_s} -\sigma _{h_s}\right) +\left( 1-\delta \right) \left( \frac{1}{\# h_s}\sum _{\imath =1}^{\#h_s}\overline{{\mathop{{\alpha _{\imath }}}\limits^{\frown}}}-\sigma _{{\mathop{{\alpha _{\imath }}}\limits^{\frown}}}\right) , \zeta \left( \overline{g_s} -\sigma _{g_s}\right) +\left( 1-\zeta \right) \left( \frac{1}{\# g_s}\sum _{\imath =1}^{\#g_s}\overline{{\mathop{{\beta _{\imath }}}\limits^{\frown}}}-\sigma _{{\mathop{{\beta _{\imath }}}\limits^{\frown}}}\right) \right] , \end{aligned}$$

where $\overline{{\mathop{{\alpha _{\imath }}}\limits^{\frown}}}= \frac{a^L_{\imath }+a^M_{\imath }+a^U_{\imath }}{3}$, $\overline{{\mathop{{\beta _{\imath }}}\limits^{\frown}}}= \frac{b^L_{\imath }+b^M_{\imath }+b^U_{\imath }}{3}$ and $\sigma _{{\mathop{{\alpha _{\imath }}}\limits^{\frown}}}=\sqrt{\left( a^L_{\imath }\right) ^2+\left( a^M_{\imath }\right) ^2+\left( a^U_{\imath }\right) ^2-a^L_{\imath }a^M_{\imath }- a^L_{\imath }a^U_{\imath }-a^M_{\imath }a^U_{\imath }}$, $\sigma _{{\mathop{{\beta _{\imath }}}\limits^{\frown}}}=\sqrt{\left( b^L_{\imath }\right) ^2+\left( b^M_{\imath }\right) ^2+\left( b^U_{\imath }\right) ^2-b^L_{\imath }b^M_{\imath }- b^L_{\imath }b^U_{\imath }-b^M_{\imath }b^U_{\imath }}$. Further, $\delta , \zeta \in (0,1)$ can be deemed as the confidence level of DMs and DMs can declare the value of $\delta$ themself freely.

According to Definition 6, the comparison rule for two NWDHFEs is summarized below.

### Definition 7

Given any two NWDHFEs $\partial _1=\left\langle h^1_s , g^1_s, \varphi \left( h^1_s \right) ,\varphi \left( g^1_s \right) \right\rangle$ and $\partial _2=\left\langle h^2_s , g^2_s , \varphi \left( h^2_s \right) ,\varphi \left( g^2_s \right) \right\rangle$, and let $S_{N_s} \left( \partial _1\right)$ and $S_{N_s} \left( \partial _2\right)$ be the score function of $\partial _1$ and $\partial _2$, respectively. Then:

1. If $$S_{N_s} \left( \partial _1\right) \ge S_{N_s} \left( \partial _2\right) $$, then $$\partial _1> \partial _2$$.
2. If $$S_{N_s} \left( \partial _1\right) \le S_{N_s} \left( \partial _2\right) $$, then $$\partial _1< \partial _2$$.
3. If $$S_{N_s} \left( \partial _1\right) =S_{N_s} \left( \partial _2\right) $$, then $$\partial _1 \sim \partial _2$$.

Here, the symbol “$\sim$” means $\partial _1$ and $\partial _2$ are indistinguishable.

## Distance and similarity measures between two NWDHFSs

Until now, there is no research on the distance and similarity measures for NWDHFSs. So, we will first propose the axioms for distance and similarity measures under dual hesitant fuzzy environment. After that, some well-known distance measures such as Hamming distance, Euclidean distance, Hausdorff distance and hybrid distance will be adopted for the definition of NWDHFSs distances.

Practically, in most of the cases, the values of number of elements in membership grade and nonmembership grade may not be equal, i.e., $\# h_s^{1}(\textit{x}_{j}) \ne \# h_s^{2}(\textit{x}_{j})$ and $\# g_s^{1}(\textit{x}_{j}) \ne \# g_s^{2}(\textit{x}_{j})$ for each $\textit{x}_{j} \in \textit{X}$. To find the distance and similarity measure between NWDHFSs, one should extend the shorter one until the membership grades and nonmembership grades of both NWDHFSs have the same length. Following the rule detailed by Xu and Zhang^63^, we can generalize it for NWDHFS setting as follows:

8 $$\begin{aligned}&\overline{h}(\textit{x}_{j})=\daleth h^{+}(\textit{x}_{j}) + (1-\daleth ) h^{-}(\textit{x}_{j}), \end{aligned}$$

9 $$\begin{aligned}&\overline{g}(\textit{x}_{j})=\gimel g^{+}(\textit{x}_{j}) + (1-\gimel ) g^{-}(\textit{x}_{j}), \end{aligned}$$

where $h^{+} (\textit{x}_{j})(g^{+}(\textit{x}_{j}))$ and $h^{-} (\textit{x}_{j})(g^{-}(\textit{x}_{j}))$ represent the largest and smallest values in each $h(\textit{x}_{j}) (g(\textit{x}_{j}))$, respectively.

To extend the shorter one, we can add any value to the shorter one according to the parameters $\daleth$ and $\gimel$. The selection of these parameters mainly depends on the DM’s risk preferences. Optimists anticipate desirable outcomes and may add the largest value of the membership grade and the smallest value of the non-membership grade, while pessimists expect unfavorable outcomes and may add the smallest of the membership grade and the largest value of the non-membership grade.

**Note:** In the current study we shall take $\daleth =\beth =1/2$.

In what follows, we state the axioms of distance and similarity measures for NWDHFSs.

### Definition 8

Let $N_1$ and $N_2$ be two NWDHFSs on $\textit{X}=\left\{ \textit{x}_1,\textit{x}_2,...,\textit{x}_n \right\}$, then the distance measure between $N_1$ and $N_2$ is defined as $d\left( N_1,N_2 \right)$ which satisfies the following properties:

1. $$0\le d\left( N_1,N_2\right) \le 1$$ ;
2. $d\left( N_1,N_2\right) =0$ if and only if $N_1=N_2$;
3. $$d\left( N_1,N_2\right) =d\left( N_2,N_1\right) $$.

### Definition 9

Let $N_1$ and $N_2$ be two NWDHFSs on $\textit{X}=\left\{ \textit{x}_1,\textit{x}_2,...,\textit{x}_n \right\}$, then the similarity measure between $N_1$ and $N_2$is defined as $\rho \left( N_1,N_2 \right)$ which satisfies the following properties:

1. $$0\le \rho \left( N_1,N_2\right) \le 1$$ ;
2. $\rho \left( N_1,N_2\right) =1$ if and only if $N_1=N_2$;
3. $$\rho \left( N_1,N_2\right) =\rho \left( N_2,N_1\right) $$.

The above-stated axioms are analogous to the axioms of distance and similarity measures for DHFSs given by^64^. These axioms are simple to comprehend, and each of them is mandatory for the definition of the measures.

Like the other FSs, the relationship between $\rho \left( N_1, N_2\right)$ and $d \left( N_1, N_2\right)$ also obeys the formulas that $\rho \left( N_1, N_2\right) = 1 - d\left( N_1, N_2\right)$. So we will mainly discuss the distances for NWDHFSs, then the similarity measures can be easily gotten.

On the basis of Definition 8, we give a normal wiggly dual hesitant normalized Hamming distance between $N_1=\left\{ \left\langle \textit{x}, h^1_s(\textit{x}), g^1_s(\textit{x}), \varphi \left( h^1_s(\textit{x}) \right) , \varphi \left( g^1_s(\textit{x}) \right) \right\rangle | \textit{x} \in X \right\}$ and $N_2=\left\{ \left\langle \textit{x}, h^2_s(\textit{x}), g^2_s(\textit{x}), \varphi \left( h^2_s(\textit{x}) \right) ,\right. \right. \left. \left. \varphi \left( g^2_s(\textit{x}) \right) \right\rangle | \textit{x} \in \textit{X} \right\}$  as:

10 $$\begin{aligned}&d\left( N_{1},N_{2} \right) \nonumber \\&\quad =\left[ \frac{1}{2n}\sum _{j=1}^{n}\left( \begin{array}{c} \frac{1}{\#h_s(\textit{x}_{j})}\left( \begin{array}{c} \sum \limits _{\imath }^{\#h_s}(\textit{x}_{j})\left| \alpha ^1_{\sigma (\imath )}(\textit{x}_{j})-\alpha ^2_{\sigma (\imath )}(\textit{x}_{j})\right| + \sum \limits _{\imath }^{\#\varphi \left( h_s\right) (\textit{x}_{j})} \left( \frac{1}{3}\left( \left| {a_{\imath }^{1}}^{L}(\textit{x}_{j})-{a_{\imath }^{2}}^{L}(\textit{x}_{j}) \right| \right. \right. \\ \left. \left. + \left| {a_{\imath }^{1}}^{M}(\textit{x}_{j})-{a_{\imath }^{2}}^{M}(\textit{x}_{j}) \right| +\left| {a_{\imath }^{1}}^{U}(\textit{x}_{j})-{a_{\imath }^{2}}^{U}(\textit{x}_{j}) \right| \right) \right) \end{array}\right) \\ + \frac{1}{\#g_s(\textit{x}_{j})}\left( \begin{array}{c} \sum \limits _{\imath }^{\#g_s}(\textit{x}_{j})\left| \beta ^1_{\sigma (\imath )}(\textit{x}_{j})-\beta ^2_{\sigma (\imath )}(\textit{x}_{j})\right| + \sum \limits _{\imath }^{\#\varphi \left( g_s\right) (\textit{x}_{j})} \left( \frac{1}{3}\left( \left| {b_{\imath }^{1}}^{L}(\textit{x}_{j})-{b_{\imath }^{2}}^{L} (\textit{x}_{j})\right| \right. \right. \\ \left. \left. +\left| {b_{\imath }^{1}}^{M}(\textit{x}_{j})-{b_{\imath }^{2}}^{M}(\textit{x}_{j}) \right| +\left| {b_{\imath }^{1}}^{U}(\textit{x}_{j})-{b_{\imath }^{2}}^{U}(\textit{x}_{j}) \right| \right) \right) \end{array}\right) \\ \end{array}\right) \right] , \end{aligned}$$

where $\alpha ^1_{\sigma (\imath )}$ and $\alpha ^2_{\sigma (\imath )}$ are the $\imath th$ largest values in $h^1_s$ and $h^2_s$ whose corresponding normal wiggly elements $\left( {a_{\imath }^{1}}^L,{a_{\imath }^{1}}^M,{a_{\imath }^{1}}^U \right)$ and $\left( {a_{\imath }^{2}}^L,{a_{\imath }^{2}}^M,{a_{\imath }^{2}}^U \right)$ respectively, while $\beta ^1_{\sigma (\imath )}$ and $\beta ^2_{\sigma (\imath )}$ are the $\imath th$ largest values in $g^1_s$ and $g^2_s$ whose corresponding normal wiggly elements $\left( {b_{\imath }^{1}}^L,{b_{\imath }^{1}}^M,{b_{\imath }^{1}}^U \right)$ and $\left( {b_{\imath }^{2}}^L,{b_{\imath }^{2}}^M,{b_{\imath }^{2}}^U \right)$, respectively.

Similarly, a dual hesitant normalized Euclidean distance can be defined as follows:

11 $$\begin{aligned}&d\left( N_{1},N_{2} \right) \nonumber \\&\quad =\left[ \frac{1}{2n}\sum _{j=1}^{n}\left( \begin{array}{c} \frac{1}{\#h_s(\textit{x}_{j})}\left( \begin{array}{c} \sum \limits _{\imath }^{\#h_s(\textit{x}_{j})}\left| \alpha ^1_{\sigma (\imath )}(\textit{x}_{j})-\alpha ^2_{\sigma (\imath )}(\textit{x}_{j})\right| ^{2} + \sum \limits _{\imath }^{\#\varphi \left( h_s\right) (\textit{x}_{j})} \left( \frac{1}{3}\left( \left| {a_{\imath }^{1}}^{L}(\textit{x}_{j})-{a_{\imath }^{2}}^{L}(\textit{x}_{j}) \right| ^2\right. \right. \\ \left. \left. +\left| {a_{\imath }^{1}}^{M}(\textit{x}_{j})-{a_{\imath }^{2}}^{M}(\textit{x}_{j}) \right| ^2 +\left| {a_{\imath }^{1}}^{U}(\textit{x}_{j})-{a_{\imath }^{2}}^{U} (x_{j})\right| ^2\right) \right) \end{array}\right) \\ + \frac{1}{\#g_s(\textit{x}_{j})}\left( \begin{array}{c} \sum \limits _{\imath }^{\#g_s(\textit{x}_{j})}\left| \beta ^1_{\sigma (\imath )}(\textit{x}_{j})-\beta ^2_{\sigma (\imath )}(\textit{x}_{j})\right| ^{2}+ \sum \limits _{\imath }^{\#\varphi \left( g_s\right) (\textit{x}_{j})} \left( \frac{1}{3}\left( \left| {b_{\imath }^{1}}^{L}(\textit{x}_{j})-{b_{\imath }^{2}}^{L}(\textit{x}_{j}) \right| ^2 \right. \right. \\ \left. \left. + \left| {b_{\imath }^{1}}^{M}(\textit{x}_{j})-{b_{\imath }^{2}}^{M}(\textit{x}_{j}) \right| ^2 +\left| {b_{\imath }^{1}}^{U}(\textit{x}_{j})-{b_{\imath }^{2}}^{U}(\textit{x}_{j}) \right| ^2\right) \right) \end{array}\right) \end{array}\right) \right] ^{1/2}. \end{aligned}$$

With the generalization of the two distances Eqs. (10) and (11), a generalized normal wiggly dual hesitant normalized distance can be obtained:

12 $$\begin{aligned}&d\left( N_{1},N_{2} \right) \nonumber \\&\quad =\left[ \frac{1}{2n}\sum _{j=1}^{n}\left( \begin{array}{c} \frac{1}{\#h_s(\textit{x}_{j})}\left( \begin{array}{c} \sum \limits _{\imath }^{\#h_s(\textit{x}_{j})}\left| \alpha ^1_{\sigma (\imath )}(\textit{x}_{j})-\alpha ^2_{\sigma (\imath )}(\textit{x}_{j})\right| ^{\lambda }+ \sum \limits _{\imath }^{\#\varphi \left( h_s\right) (\textit{x}_{j})} \left( \frac{1}{3}\left( \left| {a_{\imath }^{1}}^{L}(\textit{x}_{j})-{a_{\imath }^{2}}^{L}(\textit{x}_{j}) \right| ^{\lambda }\right. \right. \\ \left. \left. +\left| {a_{\imath }^{1}}^{M}(\textit{x}_{j})-{a_{\imath }^{2}}^{M} (\textit{x}_{j})\right| ^{\lambda } +\left| {a_{\imath }^{1}}^{U}(\textit{x}_{j})-{a_{\imath }^{2}}^{U}(\textit{x}_{j}) \right| ^{\lambda }\right) \right) \end{array}\right) \\ + \frac{1}{\#g_s(\textit{x}_{j})}\left( \begin{array}{c} \sum \limits _{\imath }^{\#g_s(\textit{x}_{j})}\left| \beta ^1_{\sigma (\imath )}(\textit{x}_{j})-\beta ^2_{\sigma (\imath )}(\textit{x}_{j})\right| ^{\lambda }+ \sum \limits _{\imath }^{\#\varphi \left( g_s(\textit{x}_{j})\right) } \left( \frac{1}{3}\left( \left| {b_{\imath }^{1}}^{L}(\textit{x}_{j})-{b_{\imath }^{2}}^{L}(\textit{x}_{j}) \right| ^{\lambda }\right. \right. \\ \left. \left. +\left| {b_{\imath }^{1}}^{M}(\textit{x}_{j})-{b_{\imath }^{2}}^{M}(\textit{x}_{j}) \right| ^{\lambda } +\left| {b_{\imath }^{1}}^{U}(\textit{x}_{j})-{b_{\imath }^{2}}^{U}(\textit{x}_{j}) \right| ^{\lambda }\right) \right) \end{array}\right) \end{array}\right) \right] ^{1/\lambda }. \end{aligned}$$

Analogously, the Hausdorff distance measure can be proposed for NWHFSs, for two NWHFSs $N_1$ and $N_2$, the generalized normal wiggly dual hesitant normalized Hausdorff distance measure can be defined as:

13 $$\begin{aligned}&d\left( N_{1},N_{2} \right) \nonumber \\&\quad =\left[ \frac{1}{n}\sum _{j=1}^{n} \max \left( \begin{array}{c} \max \limits _{\imath }\left( \begin{array}{c} \left| \alpha ^1_{\sigma (\imath )}(\textit{x}_{j})-\alpha ^2_{\sigma (\imath )}(\textit{x}_{j})\right| ^{\lambda }+ \left( \frac{1}{3}\left( \left| {a_{\imath }^{1}}^{L}(\textit{x}_{j})-{a_{\imath }^{2}}^{L}(\textit{x}_{j}) \right| ^{\lambda }\right. \right. \\ \left. \left. +\left| {a_{\imath }^{1}}^{M}(\textit{x}_{j})-{a_{\imath }^{2}}^{M}(\textit{x}_{j}) \right| ^{\lambda } +\left| {a_{\imath }^{1}}^{U}(\textit{x}_{j})-{a_{\imath }^{2}}^{U}(\textit{x}_{j}) \right| ^{\lambda }\right) \right) \end{array}\right) , \\ \max \limits _{k}\left( \begin{array}{c} \left| \beta ^1_{\sigma (k)}(\textit{x}_{j})-\beta ^2_{\sigma (k)}(\textit{x}_{j})\right| ^{\lambda }+ \left( \frac{1}{3}\left( \left| {b_{k}^{1}}^{L}(\textit{x}_{j})-{b_{k}^{2}}^{L}(\textit{x}_{j}) \right| ^{\lambda }\right. \right. \\ \left. \left. +\left| {b_{k}^{1}}^{M}(\textit{x}_{j})-{b_{k}^{2}}^{M}(\textit{x}_{j}) \right| ^{\lambda } +\left| {b_{k}^{1}}^{U}(\textit{x}_{j})-{b_{k}^{2}}^{U}(\textit{x}_{j}) \right| ^{\lambda }\right) \right) \end{array}\right) \end{array}\right) \right] ^{1/\lambda }. \end{aligned}$$

In particular, if $\lambda =1$, then the above generalized normal wiggly dual hesitant normalized Hausdorff distance reduces to normal wiggly dual hesitant normalized Hamming-Hausdorff distance:

14 $$\begin{aligned}&d\left( N_{1},N_{2} \right) \nonumber \\&\quad =\left[ \frac{1}{n}\sum _{j=1}^{n} \max \left( \begin{array}{c} \max \limits _{\imath }\left( \begin{array}{c} \left| \alpha ^1_{\sigma (\imath )}(\textit{x}_{j})-\alpha ^2_{\sigma (\imath )}(\textit{x}_{j})\right| + \left( \frac{1}{3}\left( \left| {a_{\imath }^{1}}^{L}(\textit{x}_{j})-{a_{\imath }^{2}}^{L}(\textit{x}_{j}) \right| \right. \right. \\ \left. \left. +\left| {a_{\imath }^{1}}^{M}(\textit{x}_{j})-{a_{\imath }^{2}}^{M}(\textit{x}_{j}) \right| +\left| {a_{\imath }^{1}}^{U}(\textit{x}_{j})-{a_{\imath }^{2}}^{U}(\textit{x}_{j}) \right| \right) \right) \end{array}\right), \\  \max \limits _{k}\left( \begin{array}{c} \left| \beta ^1_{\sigma (k)}(\textit{x}_{j})-\beta ^2_{\sigma (k)}(\textit{x}_{j})\right| + \left( \frac{1}{3}\left( \left| {b_{k}^{1}}^{L}(\textit{x}_{j})-{b_{k}^{2}}^{L}(\textit{x}_{j}) \right| \right. \right. \\ \left. \left. +\left| {b_{k}^{1}}^{M}(\textit{x}_{j})-{b_{k}^{2}}^{M}(\textit{x}_{j}) \right| +\left| {b_{k}^{1}}^{U}(\textit{x}_{j})-{b_{k}^{2}}^{U}(\textit{x}_{j}) \right| \right) \right) \end{array}\right) \end{array}\right) \right] . \end{aligned}$$

If $\lambda =2$, then the above generalized normal wiggly dual hesitant normalized Hausdorff distance becomes the normal wiggly dual hesitant normalized Euclidean-Hausdorff distance:

15 $$\begin{aligned}&d\left( N_{1},N_{2} \right) \nonumber \\&\quad =\left[ \frac{1}{n}\sum _{j=1}^{n} \max \left( \begin{array}{c} \max \limits _{\imath }\left( \begin{array}{c} \left| \alpha ^1_{\sigma (\imath )}(\textit{x}_{j})-\alpha ^2_{\sigma (\imath )}(\textit{x}_{j})\right| ^{2}+ \left( \frac{1}{3}\left( \left| {a_{\imath }^{1}}^{L}(\textit{x}_{j})-{a_{\imath }^{2}}^{L}(\textit{x}_{j}) \right| ^{2}\right. \right. \\ \left. \left. +\left| {a_{\imath }^{1}}^{M}(\textit{x}_{j})-{a_{\imath }^{2}}^{M}(\textit{x}_{j}) \right| ^{2} +\left| {a_{\imath }^{1}}^{U}(\textit{x}_{j})-{a_{\imath }^{2}}^{U}(\textit{x}_{j}) \right| ^{2}\right) \right) \end{array}\right), \\ \max \limits _{k}\left( \begin{array}{c} \left| \beta ^1_{\sigma (k)}(\textit{x}_{j})-\beta ^2_{\sigma (k)}(\textit{x}_{j})\right| ^{2}+ \left( \frac{1}{3}\left( \left| {b_{k}^{1}}^{L}(\textit{x}_{j})-{b_{k}^{2}}^{L}(\textit{x}_{j}) \right| ^{2}\right. \right. \\ \left. \left. +\left| {b_{k}^{1}}^{M}(\textit{x}_{j})-{b_{k}^{2}}^{M}(\textit{x}_{j}) \right| ^{2} +\left| {b_{k}^{1}}^{U}(\textit{x}_{j})-{b_{k}^{2}}^{U}(\textit{x}_{j}) \right| ^{2}\right) \right) \end{array}\right) \end{array}\right) \right] ^{1/2}. \end{aligned}$$

Further, we can deduce a class of hybrid distance measures by combining the above distance measures, such as:

1. The hybrid normal wiggly dual hesitant normalized Hamming distance between $N_{1}$ and $N_{2}$:
  16 $$\begin{aligned}&d\left( N_{1},N_{2} \right) \nonumber \\&\quad =\frac{1}{2n}\sum _{j=1}^{n} \left[ \begin{array}{c} \left( \begin{array}{c} \frac{1}{2\#h_s(\textit{x}_{j})} \left( \begin{array}{c} \sum \limits _{\imath }^{\#h_s(\textit{x}_{j})}\left| \alpha ^1_{\sigma (\imath )}(\textit{x}_{j})-\alpha ^2_{\sigma (\imath )}(\textit{x}_{j})\right| + \sum \limits _{\imath }^{\#\varphi \left( h_s\right) (\textit{x}_{j})} \left( \frac{1}{3}\left( \left| {a_{\imath }^{1}}^{L}(\textit{x}_{j})-{a_{\imath }^{2}}^{L}(\textit{x}_{j}) \right| \right. \right. \\ \left. \left. +\left| {a_{\imath }^{1}}^{M}(\textit{x}_{j})-{a_{\imath }^{2}}^{M}(\textit{x}_{j}) \right| +\left| {a_{\imath }^{1}}^{U}(\textit{x}_{j})-{a_{\imath }^{2}}^{U}(\textit{x}_{j}) \right| \right) \right) \end{array}\right) \\ +\frac{1}{2\#g_s(\textit{x}_{j})}\left( \begin{array}{c} \sum \limits _{\imath }^{\#g_s(\textit{x}_{j})}\left| \beta ^1_{\sigma (\imath )}(\textit{x}_{j})-\beta ^2_{\sigma (\imath )}(\textit{x}_{j})\right| + \sum \limits _{\imath }^{\#\varphi \left( h_s\right) (\textit{x}_{j})} \left( \frac{1}{3}\left( \left| {a_{\imath }^{1}}^{L}(\textit{x}_{j})-{a_{\imath }^{2}}^{L}(\textit{x}_{j}) \right| \right. \right. \\ \left. \left. + \left| {b_{\imath }^{1}}^{M}(\textit{x}_{j})-{b_{\imath }^{2}}^{M}(\textit{x}_{j}) \right| +\left| {b_{\imath }^{1}}^{U}(\textit{x}_{j})-{b_{\imath }^{2}}^{U}(\textit{x}_{j}) \right| \right) \right) \end{array}\right) \end{array}\right) \\ +\max \left( \begin{array}{c} \max \limits _{\imath }\left( \begin{array}{c} \left| \alpha ^1_{\sigma (\imath )}(\textit{x}_{j})-\alpha ^2_{\sigma (\imath )}(\textit{x}_{j})\right| + \left( \frac{1}{3}\left( \left| {a_{\imath }^{1}}^{L}(\textit{x}_{j})-{a_{\imath }^{2}}^{L}(\textit{x}_{j}) \right| \right. \right. \\ \left. \left. +\left| {a_{\imath }^{1}}^{M}(\textit{x}_{j})-{a_{\imath }^{2}}^{M}(\textit{x}_{j}) \right| +\left| {a_{\imath }^{1}}^{U}(\textit{x}_{j})-{a_{\imath }^{2}}^{U}(\textit{x}_{j}) \right| \right) \right) \end{array}\right) ,\\ \max \limits _{k}\left( \begin{array}{c} \left| \beta ^1_{\sigma (k)}(\textit{x}_{j})-\beta ^2_{\sigma (k)}(\textit{x}_{j})\right| + \left( \frac{1}{3}\left( \left| {b_{k}^{1}}^{L}(\textit{x}_{j})-{b_{k}^{2}}^{L}(\textit{x}_{j}) \right| \right. \right. \\ \left. \left. +\left| {b_{k}^{1}}^{M}(\textit{x}_{j})-{b_{k}^{2}}^{M}(\textit{x}_{j}) \right| +\left| {b_{k}^{1}}^{U}(\textit{x}_{j})-{b_{k}^{2}}^{U}(\textit{x}_{j}) \right| \right) \right) \end{array}\right) \end{array}\right) \end{array}\right] . \end{aligned}$$
2. The hybrid normal wiggly dual hesitant normalized Euclidean distance between $N_{1}$ and $N_{2}$:
  17 $$\begin{aligned}&d\left( N_{1},N_{2} \right) \nonumber \\&\quad =\left\{ \frac{1}{2n}\sum _{j=1}^{n} \left[ \begin{array}{c} \left( \begin{array}{c} \frac{1}{2\#h_s(\textit{x}_{j})} \left( \begin{array}{c} \sum \limits _{\imath }^{\#h_s(\textit{x}_{j})}\left| \alpha ^1_{\sigma (\imath )}(\textit{x}_{j})-\alpha ^2_{\sigma (\imath )}(\textit{x}_{j})\right| ^2 + \sum \limits _{\imath }^{\#\varphi \left( h_s\right) (\textit{x}_{j})} \left( \frac{1}{3}\left( \left| {a_{\imath }^{1}}^{L}(\textit{x}_{j})-{a_{\imath }^{2}}^{L}(\textit{x}_{j}) \right| ^2\right. \right. \\ \left. \left. +\left| {a_{\imath }^{1}}^{M}(\textit{x}_{j})-{a_{\imath }^{2}}^{M}(\textit{x}_{j}) \right| ^2 +\left| {a_{\imath }^{1}}^{U}(\textit{x}_{j})-{a_{\imath }^{2}}^{U}(\textit{x}_{j}) \right| ^2\right) \right) \end{array}\right) \\ +\frac{1}{2\#g_s(\textit{x}_{j})}\left( \begin{array}{c} \sum \limits _{\imath }^{\#g_s(\textit{x}_{j})}\left| \beta ^1_{\sigma (\imath )}(\textit{x}_{j})-\beta ^2_{\sigma (\imath )}(\textit{x}_{j})\right| ^2+ \sum \limits _{\imath }^{\#\varphi \left( h_s\right) (\textit{x}_{j})} \left( \frac{1}{3}\left( \left| {a_{\imath }^{1}}^{L}(\textit{x}_{j})-{a_{\imath }^{2}}^{L}(\textit{x}_{j}) \right| ^2\right. \right. \\ \left. \left. +\left| {b_{\imath }^{1}}^{M}(\textit{x}_{j})-{b_{\imath }^{2}}^{M}(\textit{x}_{j}) \right| ^2 +\left| {b_{\imath }^{1}}^{U}(\textit{x}_{j})-{b_{\imath }^{2}}^{U}(\textit{x}_{j}) \right| ^2\right) \right) \end{array}\right) \end{array}\right) \\ +\max \left( \begin{array}{c} \max \limits _{\imath }\left( \begin{array}{c} \left| \alpha ^1_{\sigma (\imath )}(\textit{x}_{j})-\alpha ^2_{\sigma (\imath )}(\textit{x}_{j})\right| ^2 + \left( \frac{1}{3}\left( \left| {a_{\imath }^{1}}^{L}(\textit{x}_{j})-{a_{\imath }^{2}}^{L}(\textit{x}_{j}) \right| ^2\right. \right. \\ \left. \left. +\left| {a_{\imath }^{1}}^{M}(\textit{x}_{j})-{a_{\imath }^{2}}^{M}(\textit{x}_{j}) \right| ^2 +\left| {a_{\imath }^{1}}^{U}(\textit{x}_{j})-{a_{\imath }^{2}}^{U}(\textit{x}_{j}) \right| ^2\right) \right) \end{array}\right) ,\\ \max \limits _{k}\left( \begin{array}{c} \left| \beta ^1_{\sigma (k)}(\textit{x}_{j})-\beta ^2_{\sigma (k)}(\textit{x}_{j})\right| ^2 + \left( \frac{1}{3}\left( \left| {b_{k}^{1}}^{L}(\textit{x}_{j})-{b_{k}^{2}}^{L}(\textit{x}_{j}) \right| ^2\right. \right. \\ \left. \left. +\left| {b_{k}^{1}}^{M}(\textit{x}_{j})-{b_{k}^{2}}^{M}(\textit{x}_{j}) \right| ^2 +\left| {b_{k}^{1}}^{U}(\textit{x}_{j})-{b_{k}^{2}}^{U}(\textit{x}_{j}) \right| ^2\right) \right) \end{array}\right) \end{array}\right) \end{array}\right] \right\} ^{1/2}. \end{aligned}$$
3. The generalized hybrid normal wiggly dual hesitant normalized distance between $N_{1}$ and $N_{2}$:
  18 $$\begin{aligned}&d\left( N_{1},N_{2} \right) \nonumber \\&\quad =\left\{ \frac{1}{2n}\sum _{j=1}^{n} \left[ \begin{array}{c} \left( \begin{array}{c} \frac{1}{2\#h_s(\textit{x}_{j})} \left( \begin{array}{c} \sum \limits _{\imath }^{\#h_s(\textit{x}_{j})}\left| \alpha ^1_{\sigma (\imath )}(\textit{x}_{j})-\alpha ^2_{\sigma (\imath )}(\textit{x}_{j})\right| ^{\lambda } + \sum \limits _{\imath }^{\#\varphi \left( h_s\right) (\textit{x}_{j})} \left( \frac{1}{3}\left( \left| {a_{\imath }^{1}}^{L}(\textit{x}_{j})-{a_{\imath }^{2}}^{L}(\textit{x}_{j}) \right| ^{\lambda }\right. \right. \\ \left. \left. +\left| {a_{\imath }^{1}}^{M}(\textit{x}_{j})-{a_{\imath }^{2}}^{M}(\textit{x}_{j}) \right| ^{\lambda } +\left| {a_{\imath }^{1}}^{U}(\textit{x}_{j})-{a_{\imath }^{2}}^{U}(\textit{x}_{j}) \right| ^{\lambda }\right) \right) \end{array}\right) \\ +\frac{1}{2\#g_s(\textit{x}_{j})}\left( \begin{array}{c} \sum \limits _{\imath }^{\#g_s(\textit{x}_{j})}\left| \beta ^1_{\sigma (\imath )}(\textit{x}_{j})-\beta ^2_{\sigma (\imath )}(\textit{x}_{j})\right| ^{\lambda }+ \sum \limits _{\imath }^{\#\varphi \left( h_s\right) (\textit{x}_{j})} \left( \frac{1}{3}\left( \left| {a_{\imath }^{1}}^{L}(\textit{x}_{j})-{a_{\imath }^{2}}^{L}(\textit{x}_{j}) \right| ^{\lambda }\right. \right. \\ \left. \left. + \left| {b_{\imath }^{1}}^{M}(\textit{x}_{j})-{b_{\imath }^{2}}^{M}(\textit{x}_{j}) \right| ^{\lambda } +\left| {b_{\imath }^{1}}^{U}(\textit{x}_{j})-{b_{\imath }^{2}}^{U}(\textit{x}_{j}) \right| ^{\lambda }\right) \right) \end{array}\right) \end{array}\right) \\ +\max \left( \begin{array}{c} \max \limits _{\imath }\left( \begin{array}{c} \left| \alpha ^1_{\sigma (\imath )}(\textit{x}_{j})-\alpha ^2_{\sigma (\imath )}(\textit{x}_{j})\right| ^{\lambda } + \left( \frac{1}{3}\left( \left| {a_{\imath }^{1}}^{L}(\textit{x}_{j})-{a_{\imath }^{2}}^{L}(\textit{x}_{j}) \right| ^{\lambda }\right. \right. \\ \left. \left. +\left| {a_{\imath }^{1}}^{M}(\textit{x}_{j})-{a_{\imath }^{2}}^{M}(\textit{x}_{j}) \right| ^{\lambda } +\left| {a_{\imath }^{1}}^{U}(\textit{x}_{j})-{a_{\imath }^{2}}^{U}(\textit{x}_{j}) \right| ^{\lambda }\right) \right) \end{array}\right) ,\\ \max \limits _{k}\left( \begin{array}{c} \left| \beta ^1_{\sigma (k)}(\textit{x}_{j})-\beta ^2_{\sigma (k)}(\textit{x}_{j})\right| ^{\lambda } + \left( \frac{1}{3}\left( \left| {b_{k}^{1}}^{L}(\textit{x}_{j})-{b_{k}^{2}}^{L}(\textit{x}_{j}) \right| ^{\lambda }\right. \right. \\ \left. \left. +\left| {b_{k}^{1}}^{M}(\textit{x}_{j})-{b_{k}^{2}}^{M}(\textit{x}_{j}) \right| ^{\lambda } +\left| {b_{k}^{1}}^{U}(\textit{x}_{j})-{b_{k}^{2}}^{U}(\textit{x}_{j}) \right| ^{\lambda }\right) \right) \end{array}\right) \end{array}\right) \end{array}\right] \right\} ^{1/\lambda }. \end{aligned}$$

## Weighted distance and similarity measures between two NWDHFSs

In practice, because each $\textit{x}_{\jmath }$ plays a different role in set $\textit{X}$, it should be weighted variously. Therefore, in this part, we are going to propose some weighted versions of the aforementioned distance measures.

### Weighted distance and similarity measures between two NWDHFSs in discrete case

Suppose that the weights of the elements $\textit{x}_{\jmath }(\jmath =1,2,\ldots , n)$ are $w_{\jmath }(\jmath =1,2,\ldots ,n)$, with $w_{\jmath } \in [0,1]$ and $\sum _{\imath =1}^{n} w_{\jmath } =1$. First of all, the normalized hamming distances, the normalized Euclidean distances and the normalized Hausdorff distances can be rewritten as the weighted distances, such as generalized normal wiggly dual hesitant weighted distance between $N_1$ and $N_2$ is defined as:

19 $$\begin{aligned}&d\left( N_{1},N_{2} \right) \nonumber \\&\quad =\left[ \sum _{j=1}^{n}w_j\left( \begin{array}{c} \frac{1}{2\#h_s(\textit{x}_{j})}\left( \begin{array}{c} \sum \limits _{\imath }^{\#h_s(\textit{x}_{j})}\left| \alpha ^1_{\sigma (\imath )}(\textit{x}_{j})-\alpha ^2_{\sigma (\imath )}(\textit{x}_{j})\right| ^{\lambda }+ \sum \limits _{\imath }^{\#\varphi \left( h_s\right) (\textit{x}_{j})} \left( \frac{1}{3}\left( \left| {a_{\imath }^{1}}^{L}(\textit{x}_{j})-{a_{\imath }^{2}}^{L}(\textit{x}_{j}) \right| ^{\lambda }\right. \right. \\ \left. \left. +\left| {a_{\imath }^{1}}^{M}(\textit{x}_{j})-{a_{\imath }^{2}}^{M} (\textit{x}_{j})\right| ^{\lambda } +\left| {a_{\imath }^{1}}^{U}(\textit{x}_{j})-{a_{\imath }^{2}}^{U}(\textit{x}_{j}) \right| ^{\lambda }\right) \right) \end{array}\right) \\ + \frac{1}{2\#g_s(\textit{x}_{j})}\left( \begin{array}{c} \sum \limits _{\imath }^{\#g_s(\textit{x}_{j})}\left| \beta ^1_{\sigma (\imath )}(\textit{x}_{j})-\beta ^2_{\sigma (\imath )}(\textit{x}_{j})\right| ^{\lambda }+ \sum \limits _{\imath }^{\#\varphi \left( g_s(\textit{x}_{j})\right) } \left( \frac{1}{3}\left( \left| {b_{\imath }^{1}}^{L}(\textit{x}_{j})-{b_{\imath }^{2}}^{L}(\textit{x}_{j}) \right| ^{\lambda }\right. \right. \\ \left. \left. +\left| {b_{\imath }^{1}}^{M}(\textit{x}_{j})-{b_{\imath }^{2}}^{M}(\textit{x}_{j}) \right| ^{\lambda } +\left| {b_{\imath }^{1}}^{U}(\textit{x}_{j})-{b_{\imath }^{2}}^{U}(\textit{x}_{j}) \right| ^{\lambda }\right) \right) \end{array}\right) \end{array}\right) \right] ^{1/\lambda }. \end{aligned}$$

Generalized normal wiggly dual hesitant weighted Hausdorff distance between $N_1$ and $N_2$ is defined as:

20 $$\begin{aligned}&d\left( N_{1},N_{2} \right) \nonumber \\&\quad =\left[ \sum _{j=1}^{n}w_j \max \left( \begin{array}{c} \max \limits _{\imath }\left( \begin{array}{c} \left| \alpha ^1_{\sigma (\imath )}(\textit{x}_{j})-\alpha ^2_{\sigma (\imath )}(\textit{x}_{j})\right| ^{\lambda }+ \left( \frac{1}{3}\left( \left| {a_{\imath }^{1}}^{L}(\textit{x}_{j})-{a_{\imath }^{2}}^{L}(\textit{x}_{j}) \right| ^{\lambda }\right. \right. \\ \left. \left. +\left| {a_{\imath }^{1}}^{M}(\textit{x}_{j})-{a_{\imath }^{2}}^{M}(\textit{x}_{j}) \right| ^{\lambda } +\left| {a_{\imath }^{1}}^{U}(\textit{x}_{j})-{a_{\imath }^{2}}^{U}(\textit{x}_{j}) \right| ^{\lambda }\right) \right) \end{array}\right), \\ \max \limits _{k}\left( \begin{array}{c} \left| \beta ^1_{\sigma (k)}(\textit{x}_{j})-\beta ^2_{\sigma (k)}(\textit{x}_{j})\right| ^{\lambda }+ \left( \frac{1}{3}\left( \left| {b_{k}^{1}}^{L}(\textit{x}_{j})-{b_{k}^{2}}^{L}(\textit{x}_{j}) \right| ^{\lambda }\right. \right. \\ \left. \left. +\left| {b_{k}^{1}}^{M}(\textit{x}_{j})-{b_{k}^{2}}^{M}(\textit{x}_{j}) \right| ^{\lambda } +\left| {b_{k}^{1}}^{U}(\textit{x}_{j})-{b_{k}^{2}}^{U}(\textit{x}_{j}) \right| ^{\lambda }\right) \right) \end{array}\right) \end{array}\right) \right] ^{1/\lambda }. \end{aligned}$$

where $$\lambda > 0$$.

In particular, if $\lambda =1$, then we get the normal wiggly dual hesitant weighted Hamming distance between $N_1$ and $N_2$:

21 $$\begin{aligned}&d\left( N_{1},N_{2} \right) \nonumber \\&\quad =\left[ \sum _{j=1}^{n}w_j\left( \begin{array}{c} \frac{1}{2\#h_s(\textit{x}_{j})}\left( \begin{array}{c} \sum \limits _{\imath }^{\#h_s(\textit{x}_{j})}\left| \alpha ^1_{\sigma (\imath )}(\textit{x}_{j})-\alpha ^2_{\sigma (\imath )}(\textit{x}_{j})\right| + \sum \limits _{\imath }^{\#\varphi \left( h_s\right) (\textit{x}_{j})} \left( \frac{1}{3}\left( \left| {a_{\imath }^{1}}^{L}(\textit{x}_{j})-{a_{\imath }^{2}}^{L}(\textit{x}_{j}) \right| \right. \right. \\ \left. \left. +\left| {a_{\imath }^{1}}^{M}(\textit{x}_{j})-{a_{\imath }^{2}}^{M} (\textit{x}_{j})\right| +\left| {a_{\imath }^{1}}^{U}(\textit{x}_{j})-{a_{\imath }^{2}}^{U}(\textit{x}_{j}) \right| \right) \right) \end{array}\right) \\ + \frac{1}{2\#g_s(\textit{x}_{j})}\left( \begin{array}{c} \sum \limits _{\imath }^{\#g_s(\textit{x}_{j})}\left| \beta ^1_{\sigma (\imath )}(\textit{x}_{j})-\beta ^2_{\sigma (\imath )}(\textit{x}_{j})\right| + \sum \limits _{\imath }^{\#\varphi \left( g_s(\textit{x}_{j})\right) } \left( \frac{1}{3}\left( \left| {b_{\imath }^{1}}^{L}(\textit{x}_{j})-{b_{\imath }^{2}}^{L}(\textit{x}_{j}) \right| \right. \right. \\ \left. \left. +\left| {b_{\imath }^{1}}^{M}(\textit{x}_{j})-{b_{\imath }^{2}}^{M}(\textit{x}_{j}) \right| +\left| {b_{\imath }^{1}}^{U}(\textit{x}_{j})-{b_{\imath }^{2}}^{U}(\textit{x}_{j}) \right| \right) \right) \end{array}\right) \end{array}\right) \right] . \end{aligned}$$

The normal wiggly dual hesitant weighted Hamming-Hausdorff distance between $N_{1}$ and $N_{2}$:

22 $$\begin{aligned}&d\left( N_{1},N_{2} \right) \nonumber \\&\quad =\left[ \sum _{j=1}^{n}w_j \max \left( \begin{array}{c} \max \limits _{\imath }\left( \begin{array}{c} \left| \alpha ^1_{\sigma (\imath )}(\textit{x}_{j})-\alpha ^2_{\sigma (\imath )}(\textit{x}_{j})\right| + \left( \frac{1}{3}\left( \left| {a_{\imath }^{1}}^{L}(\textit{x}_{j})-{a_{\imath }^{2}}^{L}(\textit{x}_{j}) \right| \right. \right. \\ \left. \left. +\left| {a_{\imath }^{1}}^{M}(\textit{x}_{j})-{a_{\imath }^{2}}^{M}(\textit{x}_{j}) \right| +\left| {a_{\imath }^{1}}^{U}(\textit{x}_{j})-{a_{\imath }^{2}}^{U}(\textit{x}_{j}) \right| \right) \right), \end{array}\right)  \\ \max \limits _{k}\left( \begin{array}{c} \left| \beta ^1_{\sigma (k)}(\textit{x}_{j})-\beta ^2_{\sigma (k)}(\textit{x}_{j})\right| + \left( \frac{1}{3}\left( \left| {b_{k}^{1}}^{L}(\textit{x}_{j})-{b_{k}^{2}}^{L}(\textit{x}_{j}) \right| \right. \right. \\ \left. \left. +\left| {b_{k}^{1}}^{M}(\textit{x}_{j})-{b_{k}^{2}}^{M}(\textit{x}_{j}) \right| +\left| {b_{k}^{1}}^{U}(\textit{x}_{j})-{b_{k}^{2}}^{U}(\textit{x}_{j}) \right| \right) \right) \end{array}\right) \end{array}\right) \right] . \end{aligned}$$

If $\lambda =2$, then we get the normal wiggly dual hesitant weighted Euclidean distance between $N_{1}$ and $N_{2}$:

23 $$\begin{aligned}&d\left( N_{1},N_{2} \right) \nonumber \\&\quad =\left[ \sum _{j=1}^{n}w_j\left( \begin{array}{c} \frac{1}{2\#h_s(\textit{x}_{j})}\left( \begin{array}{c} \sum \limits _{\imath }^{\#h_s(\textit{x}_{j})}\left| \alpha ^1_{\sigma (\imath )}(\textit{x}_{j})-\alpha ^2_{\sigma (\imath )}(\textit{x}_{j})\right| ^{2}+ \sum \limits _{\imath }^{\#\varphi \left( h_s\right) (\textit{x}_{j})} \left( \frac{1}{3}\left( \left| {a_{\imath }^{1}}^{L}(\textit{x}_{j})-{a_{\imath }^{2}}^{L}(\textit{x}_{j}) \right| ^{2}\right. \right. \\ \left. \left. +\left| {a_{\imath }^{1}}^{M}(\textit{x}_{j})-{a_{\imath }^{2}}^{M} (\textit{x}_{j})\right| ^{2} +\left| {a_{\imath }^{1}}^{U}(\textit{x}_{j})-{a_{\imath }^{2}}^{U}(\textit{x}_{j}) \right| ^{2}\right) \right) \end{array}\right) \\ + \frac{1}{2\#g_s(\textit{x}_{j})}\left( \begin{array}{c} \sum \limits _{\imath }^{\#g_s(\textit{x}_{j})}\left| \beta ^1_{\sigma (\imath )}(\textit{x}_{j})-\beta ^2_{\sigma (\imath )}(\textit{x}_{j})\right| ^{2}+ \sum \limits _{\imath }^{\#\varphi \left( g_s(\textit{x}_{j})\right) } \left( \frac{1}{3}\left( \left| {b_{\imath }^{1}}^{L}(\textit{x}_{j})-{b_{\imath }^{2}}^{L}(\textit{x}_{j}) \right| ^{2}\right. \right. \\ \left. \left. +\left| {b_{\imath }^{1}}^{M}(\textit{x}_{j})-{b_{\imath }^{2}}^{M}(\textit{x}_{j}) \right| ^{2} +\left| {b_{\imath }^{1}}^{U}(\textit{x}_{j})-{b_{\imath }^{2}}^{U}(\textit{x}_{j}) \right| ^{2}\right) \right) \end{array}\right) \end{array}\right) \right] ^{1/2}. \end{aligned}$$

The normal wiggly dual hesitant weighted Euclidean-Hausdorff distance:

24 $$\begin{aligned}&d\left( N_{1},N_{2} \right) \nonumber \\&\quad =\left[ \sum _{j=1}^{n}w_{j} \max \left( \begin{array}{c} \max \limits _{\imath }\left( \begin{array}{c} \left| \alpha ^1_{\sigma (\imath )}(\textit{x}_{j})-\alpha ^2_{\sigma (\imath )}(\textit{x}_{j})\right| ^{2}+ \left( \frac{1}{3}\left( \left| {a_{\imath }^{1}}^{L}(\textit{x}_{j})-{a_{\imath }^{2}}^{L}(\textit{x}_{j}) \right| ^{2}\right. \right. \\ \left. \left. +\left| {a_{\imath }^{1}}^{M}(\textit{x}_{j})-{a_{\imath }^{2}}^{M}(\textit{x}_{j}) \right| ^{2} +\left| {a_{\imath }^{1}}^{U}(\textit{x}_{j})-{a_{\imath }^{2}}^{U}(\textit{x}_{j}) \right| ^{2}\right) \right) \end{array}\right), \\  \max \limits _{k}\left( \begin{array}{c} \left| \beta ^1_{\sigma (k)}(\textit{x}_{j})-\beta ^2_{\sigma (k)}(\textit{x}_{j})\right| ^{2}+ \left( \frac{1}{3}\left( \left| {b_{k}^{1}}^{L}(\textit{x}_{j})-{b_{k}^{2}}^{L}(\textit{x}_{j}) \right| ^{2}\right. \right. \\ \left. \left. +\left| {b_{k}^{1}}^{M}(\textit{x}_{j})-{b_{k}^{2}}^{M}(\textit{x}_{j}) \right| ^{2} +\left| {b_{k}^{1}}^{U}(\textit{x}_{j})-{b_{k}^{2}}^{U}(\textit{x}_{j}) \right| ^{2}\right) \right) \end{array}\right) \end{array}\right) \right] ^{1/2}. \end{aligned}$$

Certainly, we can construct several hybrid weighted distance measures via joining the above distance measures, such as:

1. The hybrid normal wiggly dual hesitant weighted Hamming distance between $N_1$ and $N_2$:
  25 $$\begin{aligned}&d\left( N_{1},N_{2} \right) \nonumber \\&\quad =\left\{ \sum _{j=1}^{n}w_j \left[ \begin{array}{c} \left( \begin{array}{c} \frac{1}{4\#h_s(\textit{x}_{j})} \left( \begin{array}{c} \sum \limits _{\imath }^{\#h_s(\textit{x}_{j})}\left| \alpha ^1_{\sigma (\imath )}(\textit{x}_{j})-\alpha ^2_{\sigma (\imath )}(\textit{x}_{j})\right| + \sum \limits _{\imath }^{\#\varphi \left( h_s\right) (\textit{x}_{j})} \left( \frac{1}{3}\left( \left| {a_{\imath }^{1}}^{L}(\textit{x}_{j})-{a_{\imath }^{2}}^{L}(\textit{x}_{j}) \right| +\right. \right. \\ \left. \left. \left| {a_{\imath }^{1}}^{M}(\textit{x}_{j})-{a_{\imath }^{2}}^{M}(\textit{x}_{j}) \right| +\left| {a_{\imath }^{1}}^{U}(\textit{x}_{j})-{a_{\imath }^{2}}^{U}(\textit{x}_{j}) \right| \right) \right) \end{array}\right) \\ +\frac{1}{4\#g_s(\textit{x}_{j})}\left( \begin{array}{c} \sum \limits _{\imath }^{\#g_s(\textit{x}_{j})}\left| \beta ^1_{\sigma (\imath )}(\textit{x}_{j})-\beta ^2_{\sigma (\imath )}(\textit{x}_{j})\right| + \sum \limits _{\imath }^{\#\varphi \left( h_s\right) (\textit{x}_{j})} \left( \frac{1}{3}\left( \left| {a_{\imath }^{1}}^{L}(\textit{x}_{j})-{a_{\imath }^{2}}^{L}(\textit{x}_{j}) \right| +\right. \right. \\ \left. \left. \left| {b_{\imath }^{1}}^{M}(\textit{x}_{j})-{b_{\imath }^{2}}^{M}(\textit{x}_{j}) \right| +\left| {b_{\imath }^{1}}^{U}(\textit{x}_{j})-{b_{\imath }^{2}}^{U}(\textit{x}_{j}) \right| \right) \right) \end{array}\right) \end{array}\right) \\ +\frac{1}{2} \max \left( \begin{array}{c} \max \limits _{\imath }\left( \begin{array}{c} \left| \alpha ^1_{\sigma (\imath )}(\textit{x}_{j})-\alpha ^2_{\sigma (\imath )}(\textit{x}_{j})\right| + \left( \frac{1}{3}\left( \left| {a_{\imath }^{1}}^{L}(\textit{x}_{j})-{a_{\imath }^{2}}^{L}(\textit{x}_{j}) \right| ^{\lambda }\right. \right. \\ \left. \left. +\left| {a_{\imath }^{1}}^{M}(\textit{x}_{j})-{a_{\imath }^{2}}^{M}(\textit{x}_{j}) \right| +\left| {a_{\imath }^{1}}^{U}(\textit{x}_{j})-{a_{\imath }^{2}}^{U}(\textit{x}_{j}) \right| \right) \right) \end{array}\right) ,\\ \max \limits _{k}\left( \begin{array}{c} \left| \beta ^1_{\sigma (k)}(\textit{x}_{j})-\beta ^2_{\sigma (k)}(\textit{x}_{j})\right| + \left( \frac{1}{3}\left( \left| {b_{k}^{1}}^{L}(\textit{x}_{j})-{b_{k}^{2}}^{L}(\textit{x}_{j}) \right| \right. \right. \\ \left. \left. +\left| {b_{k}^{1}}^{M}(\textit{x}_{j})-{b_{k}^{2}}^{M}(\textit{x}_{j}) \right| +\left| {b_{k}^{1}}^{U}(\textit{x}_{j})-{b_{k}^{2}}^{U}(\textit{x}_{j}) \right| \right) \right) \end{array}\right) \end{array}\right) \end{array}\right] \right\} . \end{aligned}$$
2. The hybrid normal wiggly dual hesitant weighted Euclidean distance between $N_{1}$ and $N_{2}$:
  26 $$\begin{aligned}&d\left( N_{1},N_{2} \right) \nonumber \\&\quad =\left\{ \sum _{j=1}^{n}w_j \left[ \begin{array}{c} \left( \begin{array}{c} \frac{1}{4\#h_s(\textit{x}_{j})} \left( \begin{array}{c} \sum \limits _{\imath }^{\#h_s(\textit{x}_{j})}\left| \alpha ^1_{\sigma (\imath )}(\textit{x}_{j})-\alpha ^2_{\sigma (\imath )}(\textit{x}_{j})\right| ^{2} + \sum \limits _{\imath }^{\#\varphi \left( h_s\right) (\textit{x}_{j})} \left( \frac{1}{3}\left( \left| {a_{\imath }^{1}}^{L}(\textit{x}_{j})-{a_{\imath }^{2}}^{L}(\textit{x}_{j}) \right| ^{2}\right. \right. \\ \left. \left. +\left| {a_{\imath }^{1}}^{M}(\textit{x}_{j})-{a_{\imath }^{2}}^{M}(\textit{x}_{j}) \right| ^{2} +\left| {a_{\imath }^{1}}^{U}(\textit{x}_{j})-{a_{\imath }^{2}}^{U}(\textit{x}_{j}) \right| ^{2}\right) \right) \end{array}\right) \\ +\frac{1}{4\#g_s(\textit{x}_{j})}\left( \begin{array}{c} \sum \limits _{\imath }^{\#g_s(\textit{x}_{j})}\left| \beta ^1_{\sigma (\imath )}(\textit{x}_{j})-\beta ^2_{\sigma (\imath )}(\textit{x}_{j})\right| ^{2}+ \sum \limits _{\imath }^{\#\varphi \left( h_s\right) (\textit{x}_{j})} \left( \frac{1}{3}\left( \left| {a_{\imath }^{1}}^{L}(\textit{x}_{j})-{a_{\imath }^{2}}^{L}(\textit{x}_{j}) \right| ^{2}\right. \right. \\ \left. \left. +\left| {b_{\imath }^{1}}^{M}(\textit{x}_{j})-{b_{\imath }^{2}}^{M}(\textit{x}_{j}) \right| ^{2} +\left| {b_{\imath }^{1}}^{U}(\textit{x}_{j})-{b_{\imath }^{2}}^{U}(\textit{x}_{j}) \right| ^{2}\right) \right) \end{array}\right) \end{array}\right) \\ +\frac{1}{2} \max \left( \begin{array}{c} \max \limits _{\imath }\left( \begin{array}{c} \left| \alpha ^1_{\sigma (\imath )}(\textit{x}_{j})-\alpha ^2_{\sigma (\imath )}(\textit{x}_{j})\right| ^{2} + \left( \frac{1}{3}\left( \left| {a_{\imath }^{1}}^{L}(\textit{x}_{j})-{a_{\imath }^{2}}^{L}(\textit{x}_{j}) \right| ^{2}\right. \right. \\ \left. \left. +\left| {a_{\imath }^{1}}^{M}(\textit{x}_{j})-{a_{\imath }^{2}}^{M}(\textit{x}_{j}) \right| ^{2} +\left| {a_{\imath }^{1}}^{U}(\textit{x}_{j})-{a_{\imath }^{2}}^{U}(\textit{x}_{j}) \right| ^{2}\right) \right) \end{array}\right) ,\\ \max \limits _{k}\left( \begin{array}{c} \left| \beta ^1_{\sigma (k)}(\textit{x}_{j})-\beta ^2_{\sigma (k)}(\textit{x}_{j})\right| ^{2} + \left( \frac{1}{3}\left( \left| {b_{k}^{1}}^{L}(\textit{x}_{j})-{b_{k}^{2}}^{L}(\textit{x}_{j}) \right| ^{2}\right. \right. \\ \left. \left. +\left| {b_{k}^{1}}^{M}(\textit{x}_{j})-{b_{k}^{2}}^{M}(\textit{x}_{j}) \right| ^{2} +\left| {b_{k}^{1}}^{U}(\textit{x}_{j})-{b_{k}^{2}}^{U}(\textit{x}_{j}) \right| ^{2}\right) \right) \end{array}\right) \end{array}\right) \end{array}\right] \right\} ^{1/2}. \end{aligned}$$
3. The generalized hybrid normal wiggly dual hesitant weighted distance between $N_{1}$ and $N_{2}$:
  27 $$\begin{aligned}&d\left( N_{1},N_{2} \right) \nonumber \\&\quad =\left\{ \sum \limits _{j=1}^{n}w_j \left[ \begin{array}{c} \left( \begin{array}{c} \frac{1}{4\#h_s(\textit{x}_{j})} \left( \begin{array}{c} \sum \limits _{\imath }^{\#h_s(\textit{x}_{j})}\left| \alpha ^1_{\sigma (\imath )}(\textit{x}_{j})-\alpha ^2_{\sigma (\imath )}(\textit{x}_{j})\right| ^{\lambda } + \sum \limits _{\imath }^{\#\varphi \left( h_s\right) (\textit{x}_{j})} \left( \frac{1}{3}\left( \left| {a_{\imath }^{1}}^{L}(\textit{x}_{j})-{a_{\imath }^{2}}^{L}(\textit{x}_{j}) \right| ^{\lambda }\right. \right. \\ \left. \left. +\left| {a_{\imath }^{1}}^{M}(\textit{x}_{j})-{a_{\imath }^{2}}^{M}(\textit{x}_{j}) \right| ^{\lambda } +\left| {a_{\imath }^{1}}^{U}(\textit{x}_{j})-{a_{\imath }^{2}}^{U}(\textit{x}_{j}) \right| ^{\lambda }\right) \right) \end{array}\right) \\ +\frac{1}{4\#g_s(\textit{x}_{j})}\left( \begin{array}{c} \sum \limits _{\imath }^{\#g_s(\textit{x}_{j})}\left| \beta ^1_{\sigma (\imath )}(\textit{x}_{j})-\beta ^2_{\sigma (\imath )}(\textit{x}_{j})\right| ^{\lambda }+ \sum \limits _{\imath }^{\#\varphi \left( h_s\right) (\textit{x}_{j})} \left( \frac{1}{3}\left( \left| {a_{\imath }^{1}}^{L}(\textit{x}_{j})-{a_{\imath }^{2}}^{L}(\textit{x}_{j}) \right| ^{\lambda }\right. \right. \\ \left. \left. +\left| {b_{\imath }^{1}}^{M}(\textit{x}_{j})-{b_{\imath }^{2}}^{M}(\textit{x}_{j}) \right| ^{\lambda } +\left| {b_{\imath }^{1}}^{U}(\textit{x}_{j})-{b_{\imath }^{2}}^{U}(\textit{x}_{j}) \right| ^{\lambda }\right) \right) \end{array}\right) \end{array}\right) \\ +\frac{1}{2} \max \left( \begin{array}{c} \max \limits _{\imath }\left( \begin{array}{c} \left| \alpha ^1_{\sigma (\imath )}(\textit{x}_{j})-\alpha ^2_{\sigma (\imath )}(\textit{x}_{j})\right| ^{\lambda } + \left( \frac{1}{3}\left( \left| {a_{\imath }^{1}}^{L}(\textit{x}_{j})-{a_{\imath }^{2}}^{L}(\textit{x}_{j}) \right| ^{\lambda }\right. \right. \\ \left. \left. +\left| {a_{\imath }^{1}}^{M}(\textit{x}_{j})-{a_{\imath }^{2}}^{M}(\textit{x}_{j}) \right| ^{\lambda } +\left| {a_{\imath }^{1}}^{U}(\textit{x}_{j})-{a_{\imath }^{2}}^{U}(\textit{x}_{j}) \right| ^{\lambda }\right) \right) \end{array}\right) ,\\ \max \limits _{k}\left( \begin{array}{c} \left| \beta ^1_{\sigma (k)}(\textit{x}_{j})-\beta ^2_{\sigma (k)}(\textit{x}_{j})\right| ^{\lambda } + \left( \frac{1}{3}\left( \left| {b_{k}^{1}}^{L}(\textit{x}_{j})-{b_{k}^{2}}^{L}(\textit{x}_{j}) \right| ^{\lambda }\right. \right. \\ \left. \left. +\left| {b_{k}^{1}}^{M}(\textit{x}_{j})-{b_{k}^{2}}^{M}(\textit{x}_{j}) \right| ^{\lambda } +\left| {b_{k}^{1}}^{U}(\textit{x}_{j})-{b_{k}^{2}}^{U}(\textit{x}_{j}) \right| ^{\lambda }\right) \right) \end{array}\right) \end{array}\right) \end{array}\right] \right\} ^{1/\lambda }. \end{aligned}$$

### Weighted distance and similarity measures between two NWDHFSs in continuous case

In the last subsection, all the considered distance measures are based on discrete input data. However, sometimes the universe of discourse and the weights of elements are continuous. This subsection focuses on this case.

Let $\textit{x}\in [a,b]$, and the weights of $\textit{x}$ be $w(\textit{x})$, where $w(\textit{x}) \in [0,1]$ and $\int _{a}^{b}w(\textit{x})dx=1$. Then, the continuous normal wiggly dual hesitant weighted Hamming distance, the continuous normal wiggly dual hesitant weighted Euclidean distance and the generalized continuous normal wiggly dual hesitant weighted distance between $N_1$ and $N_2$ are derived as follows:

28 $$\begin{aligned}&d\left( N_{1},N_{2} \right) \nonumber \\&\quad =\int \limits _{a}^{b}w(\textit{x})\left( \begin{array}{c} \frac{1}{2\#h_s(\textit{x}_{j})}\left( \begin{array}{c} \sum \limits _{\imath }^{\#h_s(x_{j})}\left| \alpha ^1_{\sigma (\imath )}(\textit{x}_{j})-\alpha ^2_{\sigma (\imath )}(\textit{x}_{j})\right| + \sum \limits _{\imath }^{\#\varphi \left( h_s\right) (\textit{x}_{j})} \left( \frac{1}{3}\left( \left| {a_{\imath }^{1}}^{L}(\textit{x}_{j})-{a_{\imath }^{2}}^{L}(\textit{x}_{j}) \right| \right. \right. \\ \left. \left. +\left| {a_{\imath }^{1}}^{M}(\textit{x}_{j})-{a_{\imath }^{2}}^{M} (\textit{x}_{j})\right| + \left| {a_{\imath }^{1}}^{U}(\textit{x}_{j})-{a_{\imath }^{2}}^{U}(\textit{x}_{j}) \right| \right) \right) \end{array}\right) \\ + \frac{1}{2\#g_s(\textit{x}_{j})}\left( \begin{array}{c} \sum \limits _{\imath }^{\#g_s(\textit{x}_{j})}\left| \beta ^1_{\sigma (\imath )}(\textit{x}_{j})-\beta ^2_{\sigma (\imath )}(\textit{x}_{j})\right| + \sum \limits _{\imath }^{\#\varphi \left( g_s(\textit{x}_{j})\right) } \left( \frac{1}{3}\left( \left| {b_{\imath }^{1}}^{L}(\textit{x}_{j})-{b_{\imath }^{2}}^{L}(\textit{x}_{j}) \right| \right. \right. \\ \left. \left. + \left| {b_{\imath }^{1}}^{M}(\textit{x}_{j})-{b_{\imath }^{2}}^{M}(\textit{x}_{j}) \right| +\left| {b_{\imath }^{1}}^{U}(\textit{x}_{j})-{b_{\imath }^{2}}^{U}(\textit{x}_{j}) \right| \right) \right) \end{array}\right) \end{array}\right) dx, \end{aligned}$$

29 $$\begin{aligned}&d\left( N_{1},N_{2} \right) \nonumber \\&\quad =\left[ \begin{array}{c} \int \limits _{a}^{b}w(\textit{x})\left( \begin{array}{c} \frac{1}{2\#h_s(\textit{x}_{j})}\left( \begin{array}{c} \sum \limits _{\imath }^{\#h_s(x_{j})}\left| \alpha ^1_{\sigma (\imath )}(\textit{x}_{j})-\alpha ^2_{\sigma (\imath )}(\textit{x}_{j})\right| ^2+ \sum \limits _{\imath }^{\#\varphi \left( h_s\right) (\textit{x}_{j})} \left( \frac{1}{3}\left( \left| {a_{\imath }^{1}}^{L}(\textit{x}_{j})-{a_{\imath }^{2}}^{L}(\textit{x}_{j}) \right| ^2\right. \right. \\ \left. \left. +\left| {a_{\imath }^{1}}^{M}(\textit{x}_{j})-{a_{\imath }^{2}}^{M} (\textit{x}_{j})\right| ^2 + \left| {a_{\imath }^{1}}^{U}(\textit{x}_{j})-{a_{\imath }^{2}}^{U}(\textit{x}_{j}) \right| ^2\right) \right) \end{array}\right) \\ + \frac{1}{2\#g_s(\textit{x}_{j})}\left( \begin{array}{c} \sum \limits _{\imath }^{\#g_s(\textit{x}_{j})}\left| \beta ^1_{\sigma (\imath )}(\textit{x}_{j})-\beta ^2_{\sigma (\imath )}(\textit{x}_{j})\right| ^2+ \sum \limits _{\imath }^{\#\varphi \left( g_s(\textit{x}_{j})\right) } \left( \frac{1}{3}\left( \left| {b_{\imath }^{1}}^{L}(\textit{x}_{j})-{b_{\imath }^{2}}^{L}(\textit{x}_{j}) \right| ^2\right. \right. \\ \left. \left. + \left| {b_{\imath }^{1}}^{M}(\textit{x}_{j})-{b_{\imath }^{2}}^{M}(\textit{x}_{j}) \right| ^2 +\left| {b_{\imath }^{1}}^{U}(\textit{x}_{j})-{b_{\imath }^{2}}^{U}(\textit{x}_{j}) \right| ^2\right) \right) \end{array}\right) \end{array}\right) dx \end{array}\right] ^{1/2}, \end{aligned}$$

30 $$\begin{aligned}&d\left( N_{1},N_{2} \right) \nonumber \\&\quad =\left[ \begin{array}{c} \int \limits _{a}^{b}w(\textit{x})\left( \begin{array}{c} \frac{1}{2\#h_s(\textit{x}_{j})}\left( \begin{array}{c} \sum \limits _{\imath }^{\#h_s(x_{j})}\left| \alpha ^1_{\sigma (\imath )}(\textit{x}_{j})-\alpha ^2_{\sigma (\imath )}(\textit{x}_{j})\right| ^{\lambda }+ \sum \limits _{\imath }^{\#\varphi \left( h_s\right) (\textit{x}_{j})} \left( \frac{1}{3}\left( \left| {a_{\imath }^{1}}^{L}(\textit{x}_{j})-{a_{\imath }^{2}}^{L}(\textit{x}_{j}) \right| ^{\lambda }\right. \right. \\ \left. \left. +\left| {a_{\imath }^{1}}^{M}(\textit{x}_{j})-{a_{\imath }^{2}}^{M} (\textit{x}_{j})\right| ^{\lambda } + \left| {a_{\imath }^{1}}^{U}(\textit{x}_{j})-{a_{\imath }^{2}}^{U}(\textit{x}_{j}) \right| ^{\lambda }\right) \right) \end{array}\right) \\ + \frac{1}{2\#g_s(\textit{x}_{j})}\left( \begin{array}{c} \sum \limits _{\imath }^{\#g_s(\textit{x}_{j})}\left| \beta ^1_{\sigma (\imath )}(\textit{x}_{j})-\beta ^2_{\sigma (\imath )}(\textit{x}_{j})\right| ^{\lambda }+ \sum \limits _{\imath }^{\#\varphi \left( g_s(\textit{x}_{j})\right) } \left( \frac{1}{3}\left( \left| {b_{\imath }^{1}}^{L}(\textit{x}_{j})-{b_{\imath }^{2}}^{L}(\textit{x}_{j}) \right| ^{\lambda }\right. \right. \\ \left. \left. + \left| {b_{\imath }^{1}}^{M}(\textit{x}_{j})-{b_{\imath }^{2}}^{M}(\textit{x}_{j}) \right| ^{\lambda } +\left| {b_{\imath }^{1}}^{U}(\textit{x}_{j})-{b_{\imath }^{2}}^{U}(\textit{x}_{j}) \right| ^{\lambda }\right) \right) \end{array}\right) \end{array}\right) dx \end{array}\right] ^{1/\lambda }, \end{aligned}$$

where $$\lambda > 0$$.

If $w(x)=1/b-a \;\forall \; x \in [a,b]$, then Eqs. (28)–(30) reduce to the continuous normal wiggly dual hesitant normalized Hamming distance, the continuous normal wiggly dual hesitant normalized Euclidean distance and the generalized continuous normal wiggly dual hesitant normalized distance between $N_1$ and $N_2$, which are shown as follows:

31 $$\begin{aligned}&d\left( N_{1},N_{2} \right) \nonumber \\&\quad =\frac{1}{b-a}\int \limits _{a}^{b}\left( \begin{array}{c} \frac{1}{2\#h_s(\textit{x}_{j})}\left( \begin{array}{c} \sum \limits _{\imath }^{\#h_s(x_{j})}\left| \alpha ^1_{\sigma (\imath )}(\textit{x}_{j})-\alpha ^2_{\sigma (\imath )}(\textit{x}_{j})\right| + \sum \limits _{\imath }^{\#\varphi \left( h_s\right) (\textit{x}_{j})} \left( \frac{1}{3}\left( \left| {a_{\imath }^{1}}^{L}(\textit{x}_{j})-{a_{\imath }^{2}}^{L}(\textit{x}_{j}) \right| \right. \right. \\ \left. \left. +\left| {a_{\imath }^{1}}^{M}(\textit{x}_{j})-{a_{\imath }^{2}}^{M} (\textit{x}_{j})\right| + \left| {a_{\imath }^{1}}^{U}(\textit{x}_{j})-{a_{\imath }^{2}}^{U}(\textit{x}_{j}) \right| \right) \right) \end{array}\right) \\ + \frac{1}{2\#g_s(\textit{x}_{j})}\left( \begin{array}{c} \sum \limits _{\imath }^{\#g_s(\textit{x}_{j})}\left| \beta ^1_{\sigma (\imath )}(\textit{x}_{j})-\beta ^2_{\sigma (\imath )}(\textit{x}_{j})\right| + \sum \limits _{\imath }^{\#\varphi \left( g_s(\textit{x}_{j})\right) } \left( \frac{1}{3}\left( \left| {b_{\imath }^{1}}^{L}(\textit{x}_{j})-{b_{\imath }^{2}}^{L}(\textit{x}_{j}) \right| \right. \right. \\ \left. \left. + \left| {b_{\imath }^{1}}^{M}(\textit{x}_{j})-{b_{\imath }^{2}}^{M}(\textit{x}_{j}) \right| +\left| {b_{\imath }^{1}}^{U}(\textit{x}_{j})-{b_{\imath }^{2}}^{U}(\textit{x}_{j}) \right| \right) \right) \end{array}\right) \end{array}\right) dx, \end{aligned}$$

32 $$\begin{aligned}&d\left( N_{1},N_{2} \right) \nonumber \\&\quad =\left[ \frac{1}{b-a}\begin{array}{c} \int \limits _{a}^{b}\left( \begin{array}{c} \frac{1}{2\#h_s(\textit{x}_{j})}\left( \begin{array}{c} \sum \limits _{\imath }^{\#h_s(x_{j})}\left| \alpha ^1_{\sigma (\imath )}(\textit{x}_{j})-\alpha ^2_{\sigma (\imath )}(\textit{x}_{j})\right| ^2+ \sum \limits _{\imath }^{\#\varphi \left( h_s\right) (\textit{x}_{j})} \left( \frac{1}{3}\left( \left| {a_{\imath }^{1}}^{L}(\textit{x}_{j})-{a_{\imath }^{2}}^{L}(\textit{x}_{j}) \right| ^2\right. \right. \\ \left. \left. +\left| {a_{\imath }^{1}}^{M}(\textit{x}_{j})-{a_{\imath }^{2}}^{M} (\textit{x}_{j})\right| ^2 + \left| {a_{\imath }^{1}}^{U}(\textit{x}_{j})-{a_{\imath }^{2}}^{U}(\textit{x}_{j}) \right| ^2\right) \right) \end{array}\right) \\ + \frac{1}{2\#g_s(\textit{x}_{j})}\left( \begin{array}{c} \sum \limits _{\imath }^{\#g_s(\textit{x}_{j})}\left| \beta ^1_{\sigma (\imath )}(\textit{x}_{j})-\beta ^2_{\sigma (\imath )}(\textit{x}_{j})\right| ^2+ \sum \limits _{\imath }^{\#\varphi \left( g_s(\textit{x}_{j})\right) } \left( \frac{1}{3}\left( \left| {b_{\imath }^{1}}^{L}(\textit{x}_{j})-{b_{\imath }^{2}}^{L}(\textit{x}_{j}) \right| ^2\right. \right. \\ \left. \left. + \left| {b_{\imath }^{1}}^{M}(\textit{x}_{j})-{b_{\imath }^{2}}^{M}(\textit{x}_{j}) \right| ^2 +\left| {b_{\imath }^{1}}^{U}(\textit{x}_{j})-{b_{\imath }^{2}}^{U}(\textit{x}_{j}) \right| ^2\right) \right) \end{array}\right) \end{array}\right) dx \end{array}\right] ^{1/2}, \end{aligned}$$

33 $$\begin{aligned}&d\left( N_{1},N_{2} \right) \nonumber \\&\quad =\left[ \frac{1}{b-a}\begin{array}{c} \int \limits _{a}^{b}\left( \begin{array}{c} \frac{1}{2\#h_s(\textit{x}_{j})}\left( \begin{array}{c} \sum \limits _{\imath }^{\#h_s(x_{j})}\left| \alpha ^1_{\sigma (\imath )}(\textit{x}_{j})-\alpha ^2_{\sigma (\imath )}(\textit{x}_{j})\right| ^{\lambda }+ \sum \limits _{\imath }^{\#\varphi \left( h_s\right) (\textit{x}_{j})} \left( \frac{1}{3}\left( \left| {a_{\imath }^{1}}^{L}(\textit{x}_{j})-{a_{\imath }^{2}}^{L}(\textit{x}_{j}) \right| ^{\lambda }\right. \right. \\ \left. \left. +\left| {a_{\imath }^{1}}^{M}(\textit{x}_{j})-{a_{\imath }^{2}}^{M} (\textit{x}_{j})\right| ^{\lambda } + \left| {a_{\imath }^{1}}^{U}(\textit{x}_{j})-{a_{\imath }^{2}}^{U}(\textit{x}_{j}) \right| ^{\lambda }\right) \right) \end{array}\right) \\ + \frac{1}{2\#g_s(\textit{x}_{j})}\left( \begin{array}{c} \sum \limits _{\imath }^{\#g_s(\textit{x}_{j})}\left| \beta ^1_{\sigma (\imath )}(\textit{x}_{j})-\beta ^2_{\sigma (\imath )}(\textit{x}_{j})\right| ^{\lambda }+ \sum \limits _{\imath }^{\#\varphi \left( g_s(\textit{x}_{j})\right) } \left( \frac{1}{3}\left( \left| {b_{\imath }^{1}}^{L}(\textit{x}_{j})-{b_{\imath }^{2}}^{L}(\textit{x}_{j}) \right| ^{\lambda }\right. \right. \\ \left. \left. + \left| {b_{\imath }^{1}}^{M}(\textit{x}_{j})-{b_{\imath }^{2}}^{M}(\textit{x}_{j}) \right| ^{\lambda } +\left| {b_{\imath }^{1}}^{U}(\textit{x}_{j})-{b_{\imath }^{2}}^{U}(\textit{x}_{j}) \right| ^{\lambda }\right) \right) \end{array}\right) \end{array}\right) dx \end{array}\right] ^{1/\lambda }, \end{aligned}$$

where $$\lambda > 0$$.

Now we consider the Hausdorff metric. Similar to the above, the generalized continuous normal wiggly dual hesitant weighted distance measure, the continuous normal wiggly weighted Hamming-Hausdorff distance and the continuous normal wiggly dual hesitant weighted Euclidean-Hausdorff distance between $N_{1}$ and $N_{2}$ can obtained as follows:

34 $$\begin{aligned}&d\left( N_{1},N_{2} \right) \nonumber \\&\quad =\int \limits _{a}^{b} w(\textit{x}) \max \left( \begin{array}{c} \max _{\imath }\left( \begin{array}{c} \left| \alpha ^1_{\sigma (\imath )}(\textit{x}_{j})-\alpha ^2_{\sigma (\imath )}(\textit{x}_{j})\right| +\left( \frac{1}{3}\left( \left| {a_{\imath }^{1}}^{L}(\textit{x}_{j})-{a_{\imath }^{2}}^{L}(\textit{x}_{j}) \right|  \right. \right. \\ \left. \left. +\left| {a_{\imath }^{1}}^{M}(\textit{x}_{j})-{a_{\imath }^{2}}^{M}(\textit{x}_{j}) \right| +\left| {a_{\imath }^{1}}^{U}(\textit{x}_{j})-{a_{\imath }^{2}}^{U}(\textit{x}_{j}) \right| \right) \right) \end{array}\right), \\ \max _{k}\left( \begin{array}{c} \left| \beta ^1_{\sigma (k)}(\textit{x}_{j})-\beta ^2_{\sigma (k)}(\textit{x}_{j})\right| + \left( \frac{1}{3}\left( \left| {b_{k}^{1}}^{L}(\textit{x}_{j})-{b_{k}^{2}}^{L}(\textit{x}_{j}) \right| \right. \right. \\ \left. \left. +\left| {b_{k}^{1}}^{M}(\textit{x}_{j})-{b_{k}^{2}}^{M}(\textit{x}_{j}) \right| +\left| {b_{k}^{1}}^{U}(\textit{x}_{j})-{b_{k}^{2}}^{U}(\textit{x}_{j}) \right| \right) \right) \end{array}\right) \end{array}\right) dx, \end{aligned}$$

35 $$\begin{aligned}&d\left( N_{1},N_{2} \right) \nonumber \\&\quad =\left[ \int \limits _{a}^{b} w(\textit{x}) \max \left( \begin{array}{c} \max _{\imath }\left( \begin{array}{c} \left| \alpha ^1_{\sigma (\imath )}(\textit{x}_{j})-\alpha ^2_{\sigma (\imath )}(\textit{x}_{j})\right| ^2 +\left( \frac{1}{3}\left( \left| {a_{\imath }^{1}}^{L}(\textit{x}_{j})-{a_{\imath }^{2}}^{L}(\textit{x}_{j}) \right| ^2 \right. \right. \\ \left. \left. +\left| {a_{\imath }^{1}}^{M}(\textit{x}_{j})-{a_{\imath }^{2}}^{M}(\textit{x}_{j}) \right| ^2 +\left| {a_{\imath }^{1}}^{U}(\textit{x}_{j})-{a_{\imath }^{2}}^{U}(\textit{x}_{j}) \right| ^2\right) \right) \end{array}\right), \\ \max _{k}\left( \begin{array}{c} \left| \beta ^1_{\sigma (k)}(\textit{x}_{j})-\beta ^2_{\sigma (k)}(\textit{x}_{j})\right| ^2 + \left( \frac{1}{3}\left( \left| {b_{k}^{1}}^{L}(\textit{x}_{j})-{b_{k}^{2}}^{L}(\textit{x}_{j}) \right| ^2 \right. \right. \\ \left. \left. +\left| {b_{k}^{1}}^{M}(\textit{x}_{j})-{b_{k}^{2}}^{M}(\textit{x}_{j}) \right| ^2 +\left| {b_{k}^{1}}^{U}(\textit{x}_{j})-{b_{k}^{2}}^{U}(\textit{x}_{j}) \right| ^{2}\right) \right) \end{array}\right) \end{array}\right) dx\right] ^{1/2}, \end{aligned}$$

36 $$\begin{aligned}&d\left( N_{1},N_{2} \right) \nonumber \\&\quad =\left[ \int \limits _{a}^{b} w(\textit{x}) \max \left( \begin{array}{c} \max _{\imath }\left( \begin{array}{c} \left| \alpha ^1_{\sigma (\imath )}(\textit{x}_{j})-\alpha ^2_{\sigma (\imath )}(\textit{x}_{j})\right| ^{\lambda } +\left( \frac{1}{3}\left( \left| {a_{\imath }^{1}}^{L}(\textit{x}_{j})-{a_{\imath }^{2}}^{L}(\textit{x}_{j}) \right| ^{\lambda } \right. \right. \\ \left. \left. +\left| {a_{\imath }^{1}}^{M}(\textit{x}_{j})-{a_{\imath }^{2}}^{M}(\textit{x}_{j}) \right| ^{\lambda } +\left| {a_{\imath }^{1}}^{U}(\textit{x}_{j})-{a_{\imath }^{2}}^{U}(\textit{x}_{j}) \right| ^{\lambda }\right) \right) \end{array}\right), \\ \max _{k}\left( \begin{array}{c} \left| \beta ^1_{\sigma (k)}(\textit{x}_{j})-\beta ^2_{\sigma (k)}(\textit{x}_{j})\right| ^{\lambda } + \left( \frac{1}{3}\left( \left| {b_{k}^{1}}^{L}(\textit{x}_{j})-{b_{k}^{2}}^{L}(\textit{x}_{j}) \right| ^{\lambda } \right. \right. \\ \left. \left. +\left| {b_{k}^{1}}^{M}(\textit{x}_{j})-{b_{k}^{2}}^{M}(\textit{x}_{j}) \right| ^{\lambda } +\left| {b_{k}^{1}}^{U}(\textit{x}_{j})-{b_{k}^{2}}^{U}(\textit{x}_{j}) \right| ^{\lambda }\right) \right) \end{array}\right) \end{array}\right) dx\right] ^{1/\lambda }. \end{aligned}$$

If $w(x)=1/b-a\; \forall \; x \in [a,b]$, then Eqs. (34)–(36) reduce to

37 $$\begin{aligned}&d\left( N_{1},N_{2} \right) \nonumber \\&\quad =\frac{1}{b-a} \int \limits _{a}^{b} \max \left( \begin{array}{c} \max _{\imath }\left( \begin{array}{c} \left| \alpha ^1_{\sigma (\imath )}(\textit{x}_{j})-\alpha ^2_{\sigma (\imath )}(\textit{x}_{j})\right| +\left( \frac{1}{3}\left( \left| {a_{\imath }^{1}}^{L}(\textit{x}_{j})-{a_{\imath }^{2}}^{L}(\textit{x}_{j}) \right|  \right. \right. \\ \left. \left. +\left| {a_{\imath }^{1}}^{M}(\textit{x}_{j})-{a_{\imath }^{2}}^{M}(\textit{x}_{j}) \right| +\left| {a_{\imath }^{1}}^{U}(\textit{x}_{j})-{a_{\imath }^{2}}^{U}(\textit{x}_{j}) \right| \right) \right) \end{array}\right), \\ \max _{k}\left( \begin{array}{c} \left| \beta ^1_{\sigma (k)}(\textit{x}_{j})-\beta ^2_{\sigma (k)}(\textit{x}_{j})\right| + \left( \frac{1}{3}\left( \left| {b_{k}^{1}}^{L}(\textit{x}_{j})-{b_{k}^{2}}^{L}(\textit{x}_{j}) \right|  \right. \right. \\ \left. \left. +\left| {b_{k}^{1}}^{M}(\textit{x}_{j})-{b_{k}^{2}}^{M}(\textit{x}_{j}) \right| +\left| {b_{k}^{1}}^{U}(\textit{x}_{j})-{b_{k}^{2}}^{U}(\textit{x}_{j}) \right| \right) \right) \end{array}\right) \end{array}\right) dx, \end{aligned}$$

38 $$\begin{aligned}&d\left( N_{1},N_{2} \right) \nonumber \\&\quad =\left[ \frac{1}{b-a} \int \limits _{a}^{b} \max \left( \begin{array}{c} \max _{\imath }\left( \begin{array}{c} \left| \alpha ^1_{\sigma (\imath )}(\textit{x}_{j})-\alpha ^2_{\sigma (\imath )}(\textit{x}_{j})\right| ^2 +\left( \frac{1}{3}\left( \left| {a_{\imath }^{1}}^{L}(\textit{x}_{j})-{a_{\imath }^{2}}^{L}(\textit{x}_{j}) \right| ^2 \right. \right. \\ \left. \left. +\left| {a_{\imath }^{1}}^{M}(\textit{x}_{j})-{a_{\imath }^{2}}^{M}(\textit{x}_{j}) \right| ^2 +\left| {a_{\imath }^{1}}^{U}(\textit{x}_{j})-{a_{\imath }^{2}}^{U}(\textit{x}_{j}) \right| ^2\right) \right) \end{array}\right), \\ \max _{k}\left( \begin{array}{c} \left| \beta ^1_{\sigma (k)}(\textit{x}_{j})-\beta ^2_{\sigma (k)}(\textit{x}_{j})\right| ^2 + \left( \frac{1}{3}\left( \left| {b_{k}^{1}}^{L}(\textit{x}_{j})-{b_{k}^{2}}^{L}(\textit{x}_{j}) \right| ^2 \right. \right. \\ \left. \left. +\left| {b_{k}^{1}}^{M}(\textit{x}_{j})-{b_{k}^{2}}^{M}(\textit{x}_{j}) \right| ^2 +\left| {b_{k}^{1}}^{U}(\textit{x}_{j})-{b_{k}^{2}}^{U}(\textit{x}_{j}) \right| ^{2}\right) \right) \end{array}\right) \end{array}\right) dx\right] ^{1/2}, \end{aligned}$$

39 $$\begin{aligned}&d\left( N_{1},N_{2} \right) \nonumber \\&\quad =\left[ \frac{1}{b-a} \int \limits _{a}^{b} \max \left( \begin{array}{c} \max _{\imath }\left( \begin{array}{c} \left| \alpha ^1_{\sigma (\imath )}(\textit{x}_{j})-\alpha ^2_{\sigma (\imath )}(\textit{x}_{j})\right| ^{\lambda } +\left( \frac{1}{3}\left( \left| {a_{\imath }^{1}}^{L}(\textit{x}_{j})-{a_{\imath }^{2}}^{L}(\textit{x}_{j}) \right| ^{\lambda } \right. \right. \\ \left. \left. +\left| {a_{\imath }^{1}}^{M}(\textit{x}_{j})-{a_{\imath }^{2}}^{M}(\textit{x}_{j}) \right| ^{\lambda } +\left| {a_{\imath }^{1}}^{U}(\textit{x}_{j})-{a_{\imath }^{2}}^{U}(\textit{x}_{j}) \right| ^{\lambda }\right) \right) \end{array}\right), \\ \max _{k}\left( \begin{array}{c} \left| \beta ^1_{\sigma (k)}(\textit{x}_{j})-\beta ^2_{\sigma (k)}(\textit{x}_{j})\right| ^{\lambda } + \left( \frac{1}{3}\left( \left| {b_{k}^{1}}^{L}(\textit{x}_{j})-{b_{k}^{2}}^{L}(\textit{x}_{j}) \right| ^{\lambda }\right. \right. \\ \left. \left. \left| {b_{k}^{1}}^{M}(\textit{x}_{j})-{b_{k}^{2}}^{M}(\textit{x}_{j}) \right| ^{\lambda } +\left| {b_{k}^{1}}^{U}(\textit{x}_{j})-{b_{k}^{2}}^{U}(\textit{x}_{j}) \right| ^{\lambda }\right) \right) \end{array}\right) \end{array}\right) dx\right] ^{1/\lambda }. \end{aligned}$$

Naturally, we can obtain various hybrid continuous weighted distance measures, such as the hybrid continuous normal wiggly dual hesitant weighted hamming distance, the hybrid continuous normal wiggly dual hesitant weighted Euclidean distance measure and the generalized hybrid continuous normal wiggly dual hesitant weighted distance between $N_1$ and $N_2$ as follows:

40 $$\begin{aligned}&d\left( N_{1},N_{2} \right) \nonumber \\&\quad =\int \limits _{a}^{b} w(\textit{x}) \left[ \begin{array}{c} \left( \begin{array}{c} \frac{1}{4\#h_s(\textit{x}_{j})}\left( \begin{array}{c} \sum \limits _{\imath }^{\#h_s(\textit{x}_{j})}\left| \alpha ^1_{\sigma (\imath )}(\textit{x}_{j})-\alpha ^2_{\sigma (\imath )}(\textit{x}_{j})\right| +\sum \limits _{\imath }^{\#\varphi \left( h_s\right) }(\textit{x}_{j}) \left( \frac{1}{3}\left( \left| {a_{\imath }^{1}}^{L}(\textit{x}_{j})-{a_{\imath }^{2}}^{L}(\textit{x}_{j}) \right| \right. \right. \\ \left. \left. + \left| {a_{\imath }^{1}}^{M}(\textit{x}_{j})-{a_{\imath }^{2}}^{M}(\textit{x}_{j}) \right| +\left| {a_{\imath }^{1}}^{U}(\textit{x}_{j})-{a_{\imath }^{2}}^{U}(\textit{x}_{j}) \right| \right) \right) \end{array}\right) \\ + \frac{1}{4\#g_s(\textit{x}_{j})}\left( \begin{array}{c} \sum \limits _{\imath }^{\#g_s(\textit{x}_{j})}\left| \beta ^1_{\sigma (\imath )}(\textit{x}_{j})-\beta ^2_{\sigma (\imath )}(\textit{x}_{j})\right| +\sum \limits _{\imath }^{\#\varphi \left( g_s(\textit{x}_{j})\right) } \left( \frac{1}{3}\left( \left| {b_{\imath }^{1}}^{L}(\textit{x}_{j})-{b_{\imath }^{2}}^{L}(\textit{x}_{j}) \right| \right. \right. \\ \left. \left. +\left| {b_{\imath }^{1}}^{M}(\textit{x}_{j})-{b_{\imath }^{2}}^{M}(\textit{x}_{j}) \right| +\left| {b_{\imath }^{1}}^{U}(\textit{x}_{j})-{b_{\imath }^{2}}^{U}(\textit{x}_{j}) \right| \right) \right) \end{array}\right) \end{array}\right) \\ +\frac{1}{2}\max \left( \begin{array}{c} \max _{\imath }\left( \begin{array}{c} \left| \alpha ^1_{\sigma (\imath )}(\textit{x}_{j})-\alpha ^2_{\sigma (\imath )}(\textit{x}_{j})\right| +\left( \frac{1}{3}\left( \left| {a_{\imath }^{1}}^{L}(\textit{x}_{j})-{a_{\imath }^{2}}^{L}(\textit{x}_{j}) \right| \right. \right. \\ \left. \left. + \left| {a_{\imath }^{1}}^{M}(\textit{x}_{j})-{a_{\imath }^{2}}^{M}(\textit{x}_{j}) \right| +\left| {a_{\imath }^{1}}^{U}(\textit{x}_{j})-{a_{\imath }^{2}}^{U}(\textit{x}_{j}) \right| \right) \right) \end{array}\right), \\ \max _{k}\left( \begin{array}{c} \left| \beta ^1_{\sigma (k)}(\textit{x}_{j})-\beta ^2_{\sigma (k)}(\textit{x}_{j})\right| + \left( \frac{1}{3}\left( \left| {b_{k}^{1}}^{L}(\textit{x}_{j})-{b_{k}^{2}}^{L}(\textit{x}_{j}) \right| \right. \right. \\ \left. \left. + \left| {b_{k}^{1}}^{M}(\textit{x}_{j})-{b_{k}^{2}}^{M}(\textit{x}_{j}) \right| +\left| {b_{k}^{1}}^{U}(\textit{x}_{j})-{b_{k}^{2}}^{U}(\textit{x}_{j}) \right| \right) \right) \end{array}\right) \end{array}\right) \end{array}\right] dx, \end{aligned}$$

41 $$\begin{aligned}&d\left( N_{1},N_{2} \right) \nonumber \\&=\left\{ \begin{array}{c} \int \limits _{a}^{b} w(\textit{x}) \left[ \begin{array}{c} \left( \begin{array}{c} \frac{1}{4\#h_s(\textit{x}_{j})}\left( \begin{array}{c} \sum \limits _{\imath }^{\#h_s(\textit{x}_{j})}\left| \alpha ^1_{\sigma (\imath )}(\textit{x}_{j})-\alpha ^2_{\sigma (\imath )}(\textit{x}_{j})\right| ^{2} +\sum \limits _{\imath }^{\#\varphi \left( h_s\right) }(\textit{x}_{j}) \left( \frac{1}{3}\left( \left| {a_{\imath }^{1}}^{L}(\textit{x}_{j})-{a_{\imath }^{2}}^{L}(\textit{x}_{j}) \right| ^{2}\right. \right. \\ \left. \left. +\left| {a_{\imath }^{1}}^{M}(\textit{x}_{j})-{a_{\imath }^{2}}^{M}(\textit{x}_{j}) \right| ^{2} +\left| {a_{\imath }^{1}}^{U}(\textit{x}_{j})-{a_{\imath }^{2}}^{U}(\textit{x}_{j}) \right| ^{2}\right) \right) \end{array}\right) \\ + \frac{1}{4\#g_s(\textit{x}_{j})}\left( \begin{array}{c} \sum \limits _{\imath }^{\#g_s(\textit{x}_{j})}\left| \beta ^1_{\sigma (\imath )}(\textit{x}_{j})-\beta ^2_{\sigma (\imath )}(\textit{x}_{j})\right| ^{2} +\sum \limits _{\imath }^{\#\varphi \left( g_s(\textit{x}_{j})\right) } \left( \frac{1}{3}\left( \left| {b_{\imath }^{1}}^{L}(\textit{x}_{j})-{b_{\imath }^{2}}^{L}(\textit{x}_{j}) \right| ^{2} \right. \right. \\ \left. \left. +\left| {b_{\imath }^{1}}^{M}(\textit{x}_{j})-{b_{\imath }^{2}}^{M}(\textit{x}_{j}) \right| ^{2} +\left| {b_{\imath }^{1}}^{U}(\textit{x}_{j})-{b_{\imath }^{2}}^{U}(\textit{x}_{j}) \right| ^{2}\right) \right) \end{array}\right) \end{array}\right) \\ +\frac{1}{2}\max \left( \begin{array}{c} \max _{\imath }\left( \begin{array}{c} \left| \alpha ^1_{\sigma (\imath )}(\textit{x}_{j})-\alpha ^2_{\sigma (\imath )}(\textit{x}_{j})\right| ^{2} +\left( \frac{1}{3}\left( \left| {a_{\imath }^{1}}^{L}(\textit{x}_{j})-{a_{\imath }^{2}}^{L}(\textit{x}_{j}) \right| ^{2}\right. \right. \\ \left. \left. + \left| {a_{\imath }^{1}}^{M}(\textit{x}_{j})-{a_{\imath }^{2}}^{M}(\textit{x}_{j}) \right| ^{2} +\left| {a_{\imath }^{1}}^{U}(\textit{x}_{j})-{a_{\imath }^{2}}^{U}(\textit{x}_{j}) \right| ^{2}\right) \right) \end{array}\right) , \\ \max _{k}\left( \begin{array}{c} \left| \beta ^1_{\sigma (k)}(\textit{x}_{j})-\beta ^2_{\sigma (k)}(\textit{x}_{j})\right| ^{2} + \left( \frac{1}{3}\left( \left| {b_{k}^{1}}^{L}(\textit{x}_{j})-{b_{k}^{2}}^{L}(\textit{x}_{j}) \right| ^{2}\right. \right. \\ \left. \left. + \left| {b_{k}^{1}}^{M}(\textit{x}_{j})-{b_{k}^{2}}^{M}(\textit{x}_{j}) \right| ^{2} +\left| {b_{k}^{1}}^{U}(\textit{x}_{j})-{b_{k}^{2}}^{U}(\textit{x}_{j}) \right| ^{2}\right) \right) \end{array}\right) \end{array}\right) \end{array}\right] dx \end{array}\right\} ^{1/2}, \end{aligned}$$

42 $$\begin{aligned}&d\left( N_{1},N_{2} \right) \nonumber \\&\quad =\left\{ \begin{array}{c} \int \limits _{a}^{b} w(\textit{x}) \left[ \begin{array}{c} \left( \begin{array}{c} \frac{1}{4\#h_s(\textit{x}_{j})}\left( \begin{array}{c} \sum \limits _{\imath }^{\#h_s(\textit{x}_{j})}\left| \alpha ^1_{\sigma (\imath )}(\textit{x}_{j})-\alpha ^2_{\sigma (\imath )}(\textit{x}_{j})\right| ^{\lambda } +\sum \limits _{\imath }^{\#\varphi \left( h_s\right) }(\textit{x}_{j}) \left( \frac{1}{3}\left( \left| {a_{\imath }^{1}}^{L}(\textit{x}_{j})-{a_{\imath }^{2}}^{L}(\textit{x}_{j}) \right| ^{\lambda }\right. \right. \\ \left. \left. +\left| {a_{\imath }^{1}}^{M}(\textit{x}_{j})-{a_{\imath }^{2}}^{M}(\textit{x}_{j}) \right| ^{\lambda } +\left| {a_{\imath }^{1}}^{U}(\textit{x}_{j})-{a_{\imath }^{2}}^{U}(\textit{x}_{j}) \right| ^{\lambda }\right) \right) \end{array}\right) \\ + \frac{1}{4\#g_s(\textit{x}_{j})}\left( \begin{array}{c} \sum \limits _{\imath }^{\#g_s(\textit{x}_{j})}\left| \beta ^1_{\sigma (\imath )}(\textit{x}_{j})-\beta ^2_{\sigma (\imath )}(\textit{x}_{j})\right| ^{\lambda } +\sum \limits _{\imath }^{\#\varphi \left( g_s(\textit{x}_{j})\right) } \left( \frac{1}{3}\left( \left| {b_{\imath }^{1}}^{L}(\textit{x}_{j})-{b_{\imath }^{2}}^{L}(\textit{x}_{j}) \right| ^{\lambda } \right. \right. \\ \left. \left. +\left| {b_{\imath }^{1}}^{M}(\textit{x}_{j})-{b_{\imath }^{2}}^{M}(\textit{x}_{j}) \right| ^{\lambda } +\left| {b_{\imath }^{1}}^{U}(\textit{x}_{j})-{b_{\imath }^{2}}^{U}(\textit{x}_{j}) \right| ^{\lambda }\right) \right) \end{array}\right) \end{array}\right) \\ +\frac{1}{2}\max \left( \begin{array}{c} \max _{\imath }\left( \begin{array}{c} \left| \alpha ^1_{\sigma (\imath )}(\textit{x}_{j})-\alpha ^2_{\sigma (\imath )}(\textit{x}_{j})\right| ^{\lambda } +\left( \frac{1}{3}\left( \left| {a_{\imath }^{1}}^{L}(\textit{x}_{j})-{a_{\imath }^{2}}^{L}(\textit{x}_{j}) \right| ^{\lambda }\right. \right. \\ \left. \left. + \left| {a_{\imath }^{1}}^{M}(\textit{x}_{j})-{a_{\imath }^{2}}^{M}(\textit{x}_{j}) \right| ^{\lambda } +\left| {a_{\imath }^{1}}^{U}(\textit{x}_{j})-{a_{\imath }^{2}}^{U}(\textit{x}_{j}) \right| ^{\lambda }\right) \right) \end{array}\right), \\ \max _{k}\left( \begin{array}{c} \left| \beta ^1_{\sigma (k)}(\textit{x}_{j})-\beta ^2_{\sigma (k)}(\textit{x}_{j})\right| ^{\lambda } + \left( \frac{1}{3}\left( \left| {b_{k}^{1}}^{L}(\textit{x}_{j})-{b_{k}^{2}}^{L}(\textit{x}_{j}) \right| ^{\lambda }\right. \right. \\ \left. \left. + \left| {b_{k}^{1}}^{M}(\textit{x}_{j})-{b_{k}^{2}}^{M}(\textit{x}_{j}) \right| ^{\lambda } +\left| {b_{k}^{1}}^{U}(\textit{x}_{j})-{b_{k}^{2}}^{U}(\textit{x}_{j}) \right| ^{\lambda }\right) \right) \end{array}\right) \end{array}\right) \end{array}\right] dx \end{array}\right\} ^{1/\lambda }. \end{aligned}$$

Let $w(\textit{x})=1/b-a\; \forall \; \textit{x} \in [a,b]$, then Eqs. (40)–(42) reduce to

43 $$\begin{aligned}&d\left( N_{1},N_{2} \right) = \nonumber \\&\frac{1}{b-a} \int \limits _{a}^{b} \left[ \begin{array}{c} \left( \begin{array}{c} \frac{1}{4\#h_s(\textit{x}_{j})}\left( \begin{array}{c} \sum \limits _{\imath }^{\#h_s(\textit{x}_{j})}\left| \alpha ^1_{\sigma (\imath )}(\textit{x}_{j})-\alpha ^2_{\sigma (\imath )}(\textit{x}_{j})\right| +\sum \limits _{\imath }^{\#\varphi \left( h_s\right) }(\textit{x}_{j}) \left( \frac{1}{3}\left( \left| {a_{\imath }^{1}}^{L}(\textit{x}_{j})-{a_{\imath }^{2}}^{L}(\textit{x}_{j}) \right| \right. \right. \\ \left. \left. + \left| {a_{\imath }^{1}}^{M}(\textit{x}_{j})-{a_{\imath }^{2}}^{M}(\textit{x}_{j}) \right| +\left| {a_{\imath }^{1}}^{U}(\textit{x}_{j})-{a_{\imath }^{2}}^{U}(\textit{x}_{j}) \right| \right) \right) \end{array}\right) \\ + \frac{1}{4\#g_s(\textit{x}_{j})}\left( \begin{array}{c} \sum \limits _{\imath }^{\#g_s(\textit{x}_{j})}\left| \beta ^1_{\sigma (\imath )}(\textit{x}_{j})-\beta ^2_{\sigma (\imath )}(\textit{x}_{j})\right| +\sum \limits _{\imath }^{\#\varphi \left( g_s(\textit{x}_{j})\right) } \left( \frac{1}{3}\left( \left| {b_{\imath }^{1}}^{L}(\textit{x}_{j})-{b_{\imath }^{2}}^{L}(\textit{x}_{j}) \right| \right. \right. \\ \left. \left. +\left| {b_{\imath }^{1}}^{M}(\textit{x}_{j})-{b_{\imath }^{2}}^{M}(\textit{x}_{j}) \right| +\left| {b_{\imath }^{1}}^{U}(\textit{x}_{j})-{b_{\imath }^{2}}^{U}(\textit{x}_{j}) \right| \right) \right) \end{array}\right) \end{array}\right) \\ +\frac{1}{2}\max \left( \begin{array}{c} \max _{\imath }\left( \begin{array}{c} \left| \alpha ^1_{\sigma (\imath )}(\textit{x}_{j})-\alpha ^2_{\sigma (\imath )}(\textit{x}_{j})\right| +\left( \frac{1}{3}\left( \left| {a_{\imath }^{1}}^{L}(\textit{x}_{j})-{a_{\imath }^{2}}^{L}(\textit{x}_{j}) \right| \right. \right. \\ \left. \left. + \left| {a_{\imath }^{1}}^{M}(\textit{x}_{j})-{a_{\imath }^{2}}^{M}(\textit{x}_{j}) \right| +\left| {a_{\imath }^{1}}^{U}(\textit{x}_{j})-{a_{\imath }^{2}}^{U}(\textit{x}_{j}) \right| \right) \right) \end{array}\right), \\ \max _{k}\left( \begin{array}{c} \left| \beta ^1_{\sigma (k)}(\textit{x}_{j})-\beta ^2_{\sigma (k)}(\textit{x}_{j})\right| + \left( \frac{1}{3}\left( \left| {b_{k}^{1}}^{L}(\textit{x}_{j})-{b_{k}^{2}}^{L}(\textit{x}_{j}) \right| \right. \right. \\ \left. \left. + \left| {b_{k}^{1}}^{M}(\textit{x}_{j})-{b_{k}^{2}}^{M}(\textit{x}_{j}) \right| +\left| {b_{k}^{1}}^{U}(\textit{x}_{j})-{b_{k}^{2}}^{U}(\textit{x}_{j}) \right| \right) \right) \end{array}\right) \end{array}\right) \end{array}\right] dx, \end{aligned}$$

44 $$\begin{aligned}&d\left( N_{1},N_{2} \right) = \nonumber \\&\left\{ \begin{array}{c} \frac{1}{b-a}\int \limits _{a}^{b}\left[ \begin{array}{c} \left( \begin{array}{c} \frac{1}{4\#h_s(\textit{x}_{j})}\left( \begin{array}{c} \sum \limits _{\imath }^{\#h_s(\textit{x}_{j})}\left| \alpha ^1_{\sigma (\imath )}(\textit{x}_{j})-\alpha ^2_{\sigma (\imath )}(\textit{x}_{j})\right| ^{2} +\sum \limits _{\imath }^{\#\varphi \left( h_s\right) }(\textit{x}_{j}) \left( \frac{1}{3}\left( \left| {a_{\imath }^{1}}^{L}(\textit{x}_{j})-{a_{\imath }^{2}}^{L}(\textit{x}_{j}) \right| ^{2}\right. \right. \\ \left. \left. +\left| {a_{\imath }^{1}}^{M}(\textit{x}_{j})-{a_{\imath }^{2}}^{M}(\textit{x}_{j}) \right| ^{2} +\left| {a_{\imath }^{1}}^{U}(\textit{x}_{j})-{a_{\imath }^{2}}^{U}(\textit{x}_{j}) \right| ^{2}\right) \right) \end{array}\right) \\ + \frac{1}{4\#g_s(\textit{x}_{j})}\left( \begin{array}{c} \sum \limits _{\imath }^{\#g_s(\textit{x}_{j})}\left| \beta ^1_{\sigma (\imath )}(\textit{x}_{j})-\beta ^2_{\sigma (\imath )}(\textit{x}_{j})\right| ^{2} +\sum \limits _{\imath }^{\#\varphi \left( g_s(\textit{x}_{j})\right) } \left( \frac{1}{3}\left( \left| {b_{\imath }^{1}}^{L}(\textit{x}_{j})-{b_{\imath }^{2}}^{L}(\textit{x}_{j}) \right| ^{2} \right. \right. \\ \left. \left. +\left| {b_{\imath }^{1}}^{M}(\textit{x}_{j})-{b_{\imath }^{2}}^{M}(\textit{x}_{j}) \right| ^{2} +\left| {b_{\imath }^{1}}^{U}(\textit{x}_{j})-{b_{\imath }^{2}}^{U}(\textit{x}_{j}) \right| ^{2}\right) \right) \end{array}\right) \end{array}\right) \\ +\frac{1}{2}\max \left( \begin{array}{c} \max _{\imath }\left( \begin{array}{c} \left| \alpha ^1_{\sigma (\imath )}(\textit{x}_{j})-\alpha ^2_{\sigma (\imath )}(\textit{x}_{j})\right| ^{2} +\left( \frac{1}{3}\left( \left| {a_{\imath }^{1}}^{L}(\textit{x}_{j})-{a_{\imath }^{2}}^{L}(\textit{x}_{j}) \right| ^{2}\right. \right. \\ \left. \left. + \left| {a_{\imath }^{1}}^{M}(\textit{x}_{j})-{a_{\imath }^{2}}^{M}(\textit{x}_{j}) \right| ^{2} +\left| {a_{\imath }^{1}}^{U}(\textit{x}_{j})-{a_{\imath }^{2}}^{U}(\textit{x}_{j}) \right| ^{2}\right) \right) \end{array}\right), \\ \max _{k}\left( \begin{array}{c} \left| \beta ^1_{\sigma (k)}(\textit{x}_{j})-\beta ^2_{\sigma (k)}(\textit{x}_{j})\right| ^{2} + \left( \frac{1}{3}\left( \left| {b_{k}^{1}}^{L}(\textit{x}_{j})-{b_{k}^{2}}^{L}(\textit{x}_{j}) \right| ^{2}\right. \right. \\ \left. \left. + \left| {b_{k}^{1}}^{M}(\textit{x}_{j})-{b_{k}^{2}}^{M}(\textit{x}_{j}) \right| ^{2} +\left| {b_{k}^{1}}^{U}(\textit{x}_{j})-{b_{k}^{2}}^{U}(\textit{x}_{j}) \right| ^{2}\right) \right) \end{array}\right) \end{array}\right) \end{array}\right] dx \end{array}\right\} ^{1/2}, \end{aligned}$$

45 $$\begin{aligned}&d\left( N_{1},N_{2} \right) = \nonumber \\&\left\{ \begin{array}{c} \frac{1}{b-a}\int \limits _{a}^{b} \left[ \begin{array}{c} \left( \begin{array}{c} \frac{1}{4\#h_s(\textit{x}_{j})}\left( \begin{array}{c} \sum \limits _{\imath }^{\#h_s(\textit{x}_{j})}\left| \alpha ^1_{\sigma (\imath )}(\textit{x}_{j})-\alpha ^2_{\sigma (\imath )}(\textit{x}_{j})\right| ^{\lambda } +\sum \limits _{\imath }^{\#\varphi \left( h_s\right) }(\textit{x}_{j}) \left( \frac{1}{3}\left( \left| {a_{\imath }^{1}}^{L}(\textit{x}_{j})-{a_{\imath }^{2}}^{L}(\textit{x}_{j}) \right| ^{\lambda }\right. \right. \\ \left. \left. +\left| {a_{\imath }^{1}}^{M}(\textit{x}_{j})-{a_{\imath }^{2}}^{M}(\textit{x}_{j}) \right| ^{\lambda } +\left| {a_{\imath }^{1}}^{U}(\textit{x}_{j})-{a_{\imath }^{2}}^{U}(\textit{x}_{j}) \right| ^{\lambda }\right) \right) \end{array}\right) \\ + \frac{1}{4\#g_s(\textit{x}_{j})}\left( \begin{array}{c} \sum \limits _{\imath }^{\#g_s(\textit{x}_{j})}\left| \beta ^1_{\sigma (\imath )}(\textit{x}_{j})-\beta ^2_{\sigma (\imath )}(\textit{x}_{j})\right| ^{\lambda } +\sum \limits _{\imath }^{\#\varphi \left( g_s(\textit{x}_{j})\right) } \left( \frac{1}{3}\left( \left| {b_{\imath }^{1}}^{L}(\textit{x}_{j})-{b_{\imath }^{2}}^{L}(\textit{x}_{j}) \right| ^{\lambda } \right. \right. \\ \left. \left. +\left| {b_{\imath }^{1}}^{M}(\textit{x}_{j})-{b_{\imath }^{2}}^{M}(\textit{x}_{j}) \right| ^{\lambda } +\left| {b_{\imath }^{1}}^{U}(\textit{x}_{j})-{b_{\imath }^{2}}^{U}(\textit{x}_{j}) \right| ^{\lambda }\right) \right) \end{array}\right) \end{array}\right) \\ +\frac{1}{2}\max \left( \begin{array}{c} \max _{\imath }\left( \begin{array}{c} \left| \alpha ^1_{\sigma (\imath )}(\textit{x}_{j})-\alpha ^2_{\sigma (\imath )}(\textit{x}_{j})\right| ^{\lambda } +\left( \frac{1}{3}\left( \left| {a_{\imath }^{1}}^{L}(\textit{x}_{j})-{a_{\imath }^{2}}^{L}(\textit{x}_{j}) \right| ^{\lambda }\right. \right. \\ \left. \left. + \left| {a_{\imath }^{1}}^{M}(\textit{x}_{j})-{a_{\imath }^{2}}^{M}(\textit{x}_{j}) \right| ^{\lambda } +\left| {a_{\imath }^{1}}^{U}(\textit{x}_{j})-{a_{\imath }^{2}}^{U}(\textit{x}_{j}) \right| ^{\lambda }\right) \right) \end{array}\right), \\ \max _{k}\left( \begin{array}{c} \left| \beta ^1_{\sigma (k)}(\textit{x}_{j})-\beta ^2_{\sigma (k)}(\textit{x}_{j})\right| ^{\lambda } + \left( \frac{1}{3}\left( \left| {b_{k}^{1}}^{L}(\textit{x}_{j})-{b_{k}^{2}}^{L}(\textit{x}_{j}) \right| ^{\lambda }\right. \right. \\ \left. \left. + \left| {b_{k}^{1}}^{M}(\textit{x}_{j})-{b_{k}^{2}}^{M}(\textit{x}_{j}) \right| ^{\lambda } +\left| {b_{k}^{1}}^{U}(\textit{x}_{j})-{b_{k}^{2}}^{U}(\textit{x}_{j}) \right| ^{\lambda }\right) \right) \end{array}\right) \end{array}\right) \end{array}\right] dx \end{array}\right\} ^{1/\lambda }. \end{aligned}$$

## Application of the proposed distance (similarity) measures

In what follows, we illustrate the practicality and superiority of the proposed distance measures by addressing a practical example of the medical diagnosis.

### Practical example

To illustrate the proposed distance measures, we present a practical example concerning the medical diagnosis in this section.

Most diseases have a close resemblance with each other such as Bronchitis, influenza, Dyspnea, and pollen, because their symptoms are almost the same. Therefore, the experts should be more careful during testing. The best way is that experts should describe the symptoms grades by means of FSs rather than a crisp set. Among FSs, the NWDHFS is very suitable for describing the uncertain grades of an element to a given set and can be adapted to different sensitive evaluation problems. As discussed previously in section “Introduction”, the NWDHFS is not only a form of information representation but also an information mining tool. It takes DHFS as the original information, from which it digs the potential uncertain information of the DMs in order to get the complete evaluation information. This improves the accuracy of evaluation results and thus helps the experts to check the suspected cases more carefully and accurately. In this section, we use NWDHFSs to address the considered problem.

Assume that there are three suspected patients say $P_1$, $P_2$ and $P_3$ with the symptoms $S_1$(fever), $S_2$(cough), $S_3$(shortness of breath) and $S_4$(sore throat). The values of these symptoms are listed in Tables 2, respectively. The weight vector of these four symptoms is $w=(0.3,0.3,0.2,0.2)^T$. The ideal symptoms of the diseases $D_1$(bronchitis influenza), $D_2$(dyspnea) and $D_3$(pollen) are described in Table 3 in the form of DHFSs. The corresponding normal wiggly dual hesitant forms of the data presented in Tables 2 and 3 are derived in Tables 4 and 5, respectively, which will be utilized to seek diagnoses for suspected patients based mainly on the closest distance between diseases and each patient. Three kinds of distances (i.e., the generalized weighted normal wiggly dual hesitant fuzzy distance, the generalized weighted Hausdorff normal wiggly dual hesitant fuzzy distance, and the generalized weighted hybrid normal wiggly dual hesitant fuzzy distance) with different values of $\lambda$ are adopted. First of all, the generalized weighted normal wiggly dual hesitant fuzzy distances among patients and diseases are shown in Table 6.

Generally, the distances between each disease and patient decrease along with the value of $\lambda$. However, it is not difficult to conclude that no matter what the value of $\lambda$ is $P_1$ suffers from lethal disease $D_3$, $P_2$ is troubled with $D_2$ and $P_3$ is attacked by $D_1$, for the reason that the distance measure of $P_1$, $P_2$ and $P_3$ with nominated diseases is smallest.

Secondly, we take the generalized weighted Hausdorff normal wiggly dual hesitant fuzzy distance into account. By employing the distance Formulae (20), the distance measures between each disease and patient are derived in Table 7. It can be noticed from Table 7 that the main problems for $P_1$, $P_2$ and $P_3$ are $D_3$, $D_2$ and $D_1$, respectively. Further, in all cases($\lambda =1,2,4,6$), the results are stable.

**Figure 1:**
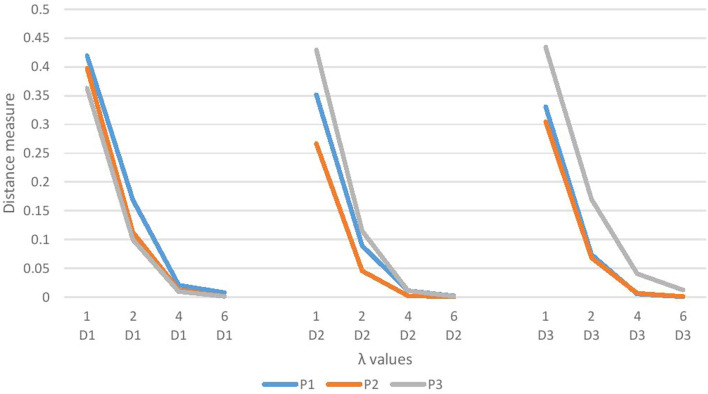
Graphical illustration of the generalized normal wiggly dual hesitant weighted distance measure.

**Figure 2:**
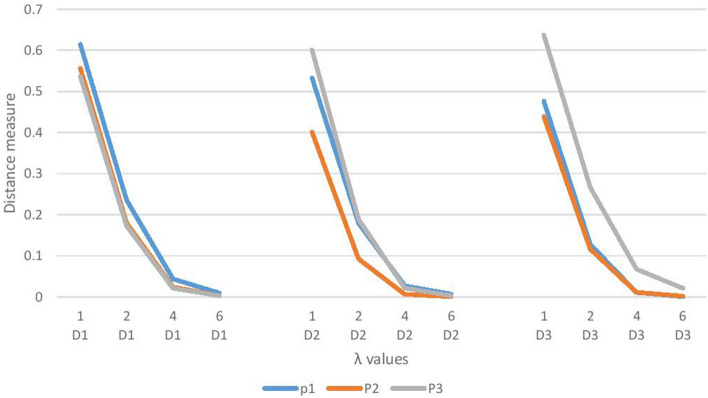
Graphical illustration of the generalized normal wiggly dual hesitant weighted Hausdorff distance measure.

**Figure 3:**
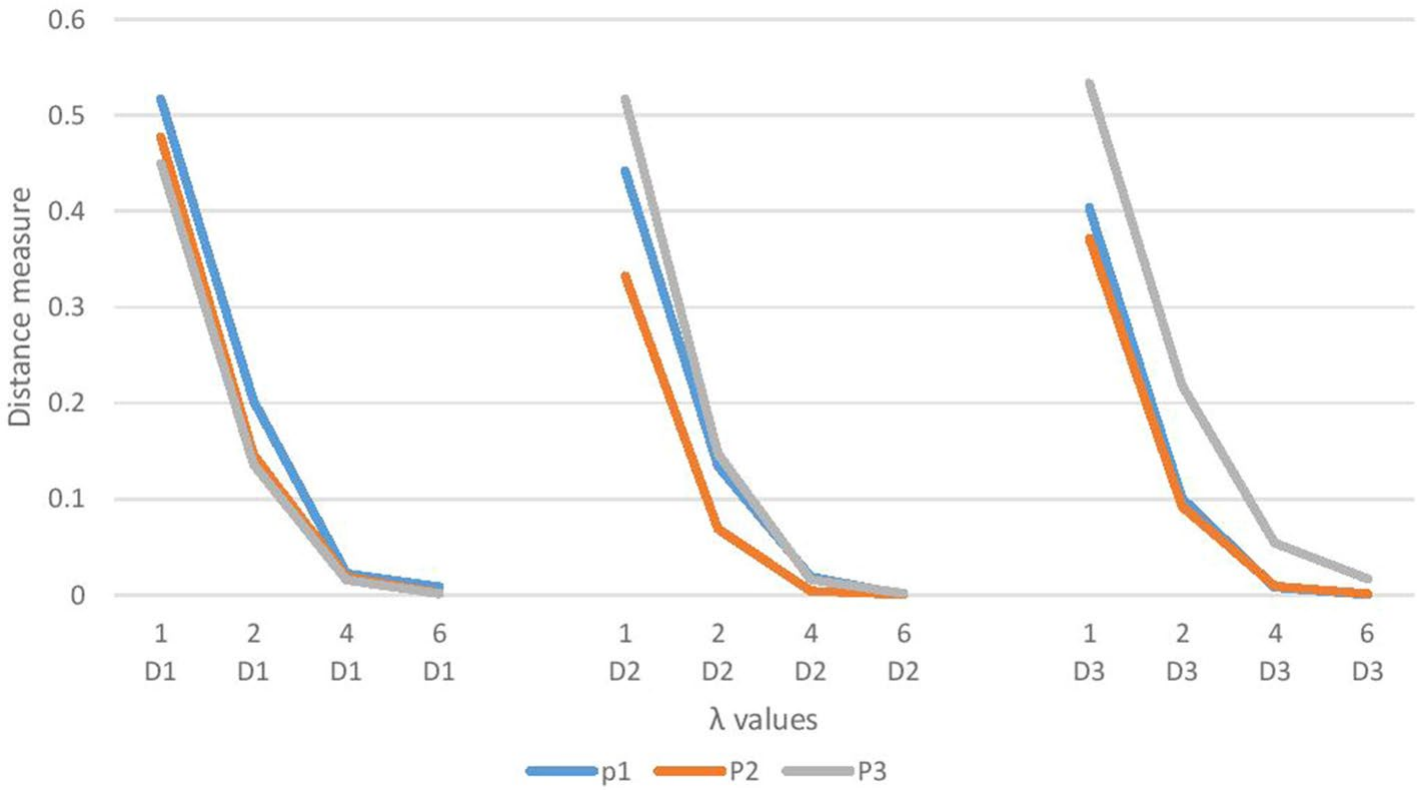
Graphical illustration of the generalized hybrid normal wiggly dual hesitant weighted distance measure.

Finally, if we employ the generalized weighted hybrid normal wiggly dual hesitant fuzzy distance to derive diagnosis for suspected patients. The obtained results are shown in Table 8. Though the formulas (19), (20) and (27) for three distance measures are different, but one can observe from Tables 6, 7, and 8 that the conclusions drawn by these formulas are same. Further, one can also observe that the computed distance measures between the patients and the diseases for $\lambda =1,2,4,6$ (but not limited to) reveal that the diagnosis remains unaltered. This finding is presented by the graphs in Figs.  1, 2 and 3, where the curves from up to down respectively in correspondence with each other. This guarantee that the proposed distance measures stability and rationality.

**Table 2:** Symptoms of three patients described with DHFSs.

	$${\varvec{s_1}}$$
$\textit{p}_1$	$\left\langle \left\{ 0.3\right\} ,\left\{ 0.6,0.5\right\} \right\rangle$
$\textit{p}_2$	$\left\langle \left\{ 0.5,0.4\right\} ,\left\{ 0.4,0.3\right\} \right\rangle$
$\textit{p}_3$	$\left\langle \left\{ 0.7,0.6\right\} ,\left\{ 0.2\right\} \right\rangle$
	$${\varvec{s_2}}$$
$\textit{p}_1$	$\left\langle \left\{ 0.7,0.6,0.5\right\} ,\left\{ 0.2,0.1 \right\} \right\rangle$
$\textit{p}_2$	$\left\langle \left\{ 0.3,0.2\right\} ,\left\{ 0.6,0.5\right\} \right\rangle$
$\textit{p}_3$	$\left\langle \left\{ 0.3,0.2\right\} ,\left\{ 0.5 \right\} \right\rangle$
	$${\varvec{s_3}}$$
$\textit{p}_1$	$\left\langle \left\{ 0.6,0.5\right\} ,\left\{ 0.1\right\} \right\rangle$
$\textit{p}_2$	$\left\langle \left\{ 0.5\right\} ,\left\{ 0.4,0.3\right\} \right\rangle$
$\textit{p}_3$	$\left\langle \left\{ 0.6,0.4\right\} ,\left\{ 0.4,0.3\right\} \right\rangle$
	$${\varvec{s_4}}$$
$\textit{p}_1$	$\left\langle \left\{ 0.3,0.2\right\} ,\left\{ 0.6\right\} \right\rangle$
$\textit{p}_2$	$\left\langle \left\{ 0.4\right\} ,\left\{ 0.3\right\} \right\rangle$
$\textit{p}_3$	$\left\langle \left\{ 0.7\right\} ,\left\{ 0.3,0.1\right\} \right\rangle$

**Table 3:** Symptoms of diseases described with DHFSs.

	$${\varvec{D_1}}$$
$\textit{s}_1$	$\left\langle \left\{ 0.5,0.4,0.3\right\} ,\left\{ 0.3,0.2\right\} \right\rangle$
$\textit{s}_2$	$\left\langle \left\{ 0.7,0.5\right\} ,\left\{ 0.2,\right\} \right\rangle$
$\textit{s}_3$	$\left\langle \left\{ 0.3,0.2\right\} ,\left\{ 0.5\right\} \right\rangle$
$\textit{s}_4$	$\left\langle \left\{ 0.7\right\} ,\left\{ 0.3,0.2\right\} \right\rangle$
	$${\varvec{D_1}}$$
$\textit{s}_1$	$\left\langle \left\{ 0.4,0.3\right\} ,\left\{ 0.5,0.3 \right\} \right\rangle$
$\textit{s}_2$	$\left\langle \left\{ 0.6,0.4,0.3\right\} ,\left\{ 0.4\right\} \right\rangle$
$\textit{s}_3$	$\left\langle \left\{ 0.4,0.3\right\} ,\left\{ 0.6,0.5 \right\} \right\rangle$
$\textit{s}_4$	$\left\langle \left\{ 0.4,0.3\right\} ,\left\{ 0.5\right\} \right\rangle$
	$${\varvec{D_1}}$$
$\textit{s}_1$	$\left\langle \left\{ 0.4,0.2\right\} ,\left\{ 0.5,0.3,0.2\right\} \right\rangle$
$\textit{s}_2$	$\left\langle \left\{ 0.4\right\} ,\left\{ 0.5,0.4\right\} \right\rangle$
$\textit{s}_3$	$\left\langle \left\{ 0.6,0.4\right\} ,\left\{ 0.3\right\} \right\rangle$
$\textit{s}_4$	$\left\langle \left\{ 0.2,0.1\right\} ,\left\{ 0.7\right\} \right\rangle$

**Table 4:** Symptoms of three patients described with NWDHFSs.

	$${\varvec{s_1}}$$
$\textit{p}_1$	$\left\langle \left\{ 0.3,0.3,0.3\right\} ,\left\{ 0.6,0.55,0.5\right\} \right.$
	$$ \left\{ (0.3,0.3,0.3),(0.3,0.3,0.3),(0.3,0.3,0.3)\right\} $$
	$$ \left. \left\{ (0.5604,0.5976,0.6396),(0.4923,0.5465,0.6077),(0.4604,0.4976,0.5396)\right\} \right\rangle $$
$\textit{p}_2$	$\left\langle \left\{ 0.5,0.45,0.4\right\} ,\left\{ 0.4,0.35,0.3\right\} \right.$
	$$ \left\{ (0.4604,0.5029,0.5396),(0.3923,0.4543,0.5077),(0.3604,0.4038,0.4396)\right\} , $$
	$$\left. \left\{ (0.3604,0.4038,0.4396),(0.2923,0.3555,0.4077),(0.2604,0.3038,0.3396)\right\} \right\rangle $$
$\textit{p}_3$	$\left\langle \left\{ 0.7,0.65,0.6\right\} ,\left\{ 0.2,0.2,0.2\right\} \right.$
	$$ \left\{ (0.6604,0.6979,0.7396),(0.5923,0.6470,0.7077),(0.5604,0.5979,0.6396)\right\} , $$
	$$\left. \left\{ (0.2,0.2,0.2),(0.2,0.2,0.2),(0.2,0.2,0.2)\right\} \right\rangle $$
	$${\varvec{s_2}}$$
$\textit{p}_1$	$\left\langle \left\{ 0.7,0.6,0.5\right\} ,\left\{ 0.2,0.1 \right\} \right.$
	$$ \left\{ (0.6206,0.6912,0.7794),(0.4845,0.5872,0.7155),(0.4206,0.4912,0.5794)\right\} ,$$
	$$\left. \left\{ (0.1697,0.2101,0.2303),(0.0697,0.1101,0.1303)\right\} \right\rangle $$
$\textit{p}_2$	$\left\langle \left\{ 0.3,0.25,0.2\right\} ,\left\{ 0.6,0.5\right\} \right.$
	$$ \left\{ (0.2604,0.3053,0.3396),(0.1923,0.2577,0.3077),(0.1604,0.2053,0.2396)\right\} $$
	$$\left. \left\{ (0.5697,0.5972,0.6303),(0.4697,0.4972,0.5303)\right\} \right\rangle $$
$\textit{p}_3$	$\left\langle \left\{ 0.3,0.25,0.2\right\} ,\left\{ 0.5,0.5 \right\} \right.$
	$$ \left\{ (0.2604,0.3053,0.3396),(0.1923,0.2577,0.3077),(0.1604,0.2053,0.2396)\right\} ,$$
	$$\left. \left\{ (0.5,0.5,0.5),(0.5,0.5,0.5)\right\} \right\rangle $$
	$${\varvec{s_3}}$$
$\textit{p}_1$	$\left\langle \left\{ 0.6,0.5\right\} ,\left\{ 0.1,0.1\right\} \right.$
	$$ \left\{ (0.5697,0.5972,0.6303),(0.4697,0.4972,0.5303)\right\} $$
	$$\left. \left\{ (0.1,0.1,0.1),(0.1,0.1,0.1)\right\} \right\rangle $$
$\textit{p}_2$	$\left\langle \left\{ 0.5,0.5\right\} ,\left\{ 0.4,0.3\right\} \right.$
	$$ \left\{ (0.5,0.5,0.5),(0.5,0.5,0.5)\right\} , $$
	$$\left. \left\{ (0.3697,0.4043,0.4303),(0.2697,0.3043,0.3303)\right\} \right\rangle $$
$\textit{p}_3$	$\left\langle \left\{ 0.6,0.4\right\} ,\left\{ 0.4,0.3\right\} \right.$
	$$ \left\{ (0.5393,0.6,0.6607),(0.3393,0.4,0.4607)\right\} ,$$
	$$\left. \left\{ (0.3697,0.4043,0.4303),(0.2697,0.3043,0.3303)\right\} \right\rangle $$
	$${\varvec{s_4}}$$
$\textit{p}_1$	$\left\langle \left\{ 0.3,0.2\right\} ,\left\{ 0.6,0.6\right\} \right.$
	$$ \left\{ (0.2697,0.3060,0.3303),(0.1697,0.2060,0.2303)\right\} $$
	$$\left. ,\left\{ (0.6,0.6,0.6),(0.6,0.6,0.6)\right\} \right\rangle $$
$\textit{p}_2$	$\left\langle \left\{ 0.4,0.4\right\} ,\left\{ 0.3,0.3\right\} \right.$
	$$ \left\{ (0.4,0.4,0.4),(0.4,0.4,0.4)\right\} ,$$
	$$ \left. \left\{ (0.3,0.3,0.3),(0.3,0.3,0.3)\right\} \right\rangle $$
$\textit{p}_3$	$\left\langle \left\{ 0.7,0.7\right\} ,\left\{ 0.3,0.1\right\} \right.$
	$$ \left\{ (0.7,0.7,0.7),(0.7,0.7,0.7)\right\} ,$$
	$$\left. \left\{ (0.2393,0.3304,0.3607),(0.0393,0.1304,0.1607)\right\} \right\rangle $$

**Table 5:** Symptoms of diseases described with NWDHFSs.

	$${\varvec{D_1}}$$
$\textit{s}_1$	$\left\langle \left\{ 0.5,0.4,0.3\right\} ,\left\{ 0.3,0.25,0.2\right\} \right.$
	$$ \left\{ (0.4615,0.5064,0.5385),(0.3184,0.4136,0.4816),(0.2615,0.3064,0.3385)\right\} $$
	$$\left. \left\{ (0.2807,0.3026,0.3193),(0.0408,0.2554,0.2908),(0.1807,0.2026,0.2193)\right\} \right\rangle $$
$\textit{s}_2$	$\left\langle \left\{ 0.7,0.6,0.5\right\} ,\left\{ 0.2,0.2\right\} \right.$
	$$ \left\{ (0.6615,0.6957,0.7385),(0.5184,0.5909,0.6816),(0.4615,0.4957,0.5385)\right\} $$
	$$\left. \left\{ (0.2,0.2,0.2),(0.2,0.2,0.2)\right\} \right\rangle $$
$\textit{s}_3$	$\left\langle \left\{ 0.3,0.2\right\} ,\left\{ 0.5,0.5\right\} \right.$
	$$ \left\{ (0.2697,0.3060,0.3303),(0.1697,0.2060,0.2303)\right\} ,$$
	$$\left. \left\{ (0.5,0.5,0.5),(0.5,0.5,0.5)\right\} \right\rangle $$
$\textit{s}_4$	$\left\langle \left\{ 0.7,0.7 \right\} ,\left\{ 0.3,0.2\right\} \right.$
	$$ \left\{ (0.7,0.7,0.7),(0.7,0.7,0.7)\right\} $$
	$$\left. \left\{ (0.2697,0.3060,0.3303),(0.1697,0.2060,0.2303)\right\} \right\rangle $$
	$${\varvec{D_2}}$$
$\textit{s}_1$	$\left\langle \left\{ 0.4,0.35,0.3\right\} ,\left\{ 0.5,0.4,0.3 \right\} \right.$
	$$ \left\{ (0.3807,0.4018,0.4193),(0.3092,0.3539,0.3908),(0.2807,0.3018,0.3193)\right\} ,$$
	$$\left. \left\{ (0.4615,0.5064,0.5385),(0.3184,0.4136,0.4816),(0.2615,0.3064,0.3385)\right\} \right\rangle $$
$\textit{s}_2$	$\left\langle \left\{ 0.6,0.4,0.3\right\} ,\left\{ 0.4,0.4\right\} \right.$
	$$ \left\{ (0.549,0.6118,0.651),(0.2797,0.4278,0.5203),(0.2296,0.3162,0.3704)\right\} ,$$
	$$ \left. \left\{ (0.4,0.4,0.4),(0.4,0.4,0.4)\right\} \right\rangle $$
$\textit{s}_3$	$\left\langle \left\{ 0.4,0.3\right\} ,\left\{ 0.6,0.5 \right\} \right.$
	$$ \left\{ (0.3697,0.4043,0.4303),(0.2697,0.3043,0.3303)\right\} ,$$
	$$ \left. \left\{ (0.5697,0.5972,0.6303),(0.4697,0.4972,0.5303)\right\} \right\rangle $$
$\textit{s}_4$	$\left\langle \left\{ 0.4,0.3\right\} ,\left\{ 0.5,0.5\right\} \right.$
	$$ \left\{ (0.3697,0.4043,0.4303),(0.2697,0.3043,0.3303)\right\} ,$$
	$$\left. \left\{ (0.5,0.5,0.5),(0.5,0.5,0.5)\right\} \right\rangle $$
	$${\varvec{D_3}}$$
$\textit{s}_1$	$\left\langle \left\{ 0.4,0.3,0.2\right\} ,\left\{ 0.5,0.3,0.2\right\} \right.$
	$$ \left\{ (0.3615,0.4085,0.4385),(0.2184,0.3181,0.3816),(0.1615,0.2085,0.2385)\right\} $$
	$$\left. \left\{ (0.449,0.5153,0.551),(0.1797,0.3360,0.4203),(0.1296,0.2211,0.2704)\right\} \right\rangle $$
$\textit{s}_2$	$\left\langle \left\{ 0.4,0.4,0.4\right\} ,\left\{ 0.5,0.4\right\} \right.$
	$$ \left\{ (0.4,0.4,0.4),(0.4,0.4,0.4),(0.4,0.4,0.4)\right\} $$
	$$\left. \left\{ (0.4697,0.5034,0.5303),(0.3697,0.4034,0.4303)\right\} \right\rangle $$
$\textit{s}_3$	$\left\langle \left\{ 0.6,0.4\right\} ,\left\{ 0.3,0.3\right\} \right.$
	$$ \left\{ (0.5393,0.6,0.6607),(0.3393,0.4,0.4607)\right\} $$
	$$ \left. \left\{ (0.3,0.3,0.3),(0.3,0.3,0.3)\right\} \right\rangle $$
$\textit{s}_4$	$\left\langle \left\{ 0.2,0.1\right\} ,\left\{ 0.7,0.7\right\} \right.$
	$$ \left\{ (0.1697,0.2101,0.2303),(0.0697,0.1101,0.1303)\right\} $$
	$$\left. \left\{ (0.7,0.7,0.7),(0.7,0.7,0.7)\right\} \right\rangle $$

**Table 6:** The generalized normal wiggly dual hesitant weighted distances among patients and diseases.

	$\lambda =1$			$\lambda =2$			$\lambda =4$			$\lambda =6$		
	$P_1$	$P_2$	$P_3$	$P_1$	$P_2$	$P_3$	$P_1$	$P_2$	$P_3$	$P_1$	$P_2$	$P_3$
$D_1$	0.4193	0.3977	**0.3629**	0.1689	0.1125	**0.0996**	0.02044	0.0123	**0.0093**	0.0076	0.0009	**0.0010**
$D_2$	0.3519	**0.2664**	0.4295	0.0889	**0.0453**	0.1145	0.0112	**0.0020**	0.0108	0.0020	**0.0001**	0.0013
$D_3$	**0.3306**	0.3046	0.4343	**0.0738**	0.0681	0.1698	**0.0052**	0.0067	0.0404	**0.0004**	0.0009	0.0120

Significant values are in bold.

**Table 7:** The generalized normal wiggly dual hesitant weighted Hausdorff distances among patients and diseases.

	$\lambda =1$			$\lambda =2$			$\lambda =4$			$\lambda =6$		
	$P_1$	$P_2$	$P_3$	$P_1$	$P_2$	$P_3$	$P_1$	$P_2$	$P_3$	$P_1$	$P_2$	$P_3$
$D_1$	0.6145	0.5562	**0.5365**	0.2356	0.1795	**0.1726**	0.0436	0.0240	**0.0209**	0.0091	0.0028	**0.0025**
$D_2$	0.5325	**0.4007**	0.6007	0.1794	**0.0925**	0.1890	0.0269	**0.0063**	0.0216	0.0068	**0.0005**	0.0026
$D_3$	**0.4770**	0.4393	0.6380	**0.1276**	0.1164	0.2668	**0.0109**	0.0114	0.0676	**0.0006**	0.002	0.0212

Significant values are in bold.

**Table 8:** The generalized hybrid normal wiggly dual hesitant weighted distances among patients and diseases.

	$\lambda =1$			$\lambda =2$			$\lambda =4$			$\lambda =6$		
	$P_1$	$P_2$	$P_3$	$P_1$	$P_2$	$P_3$	$P_1$	$P_2$	$P_3$	$P_1$	$P_2$	$P_3$
$D_1$	0.5169	0.4769	**0.4497**	0.2023	0.1460	**0.1360**	0.0221	0 .0182	**0.0154**	0.0084	0.0018	**0.0012**
$D_2$	0.4422	**0.3327**	0.5175	0.1342	**0.0689**	0.1473	0.0190	**0.0042**	0.0159	0.0005	**0.0003**	0.0018
$D_3$	**0.4038**	0.3719	0.5338	**0.1007**	0.0923	0.2183	**0.0080**	0.0090	0.0540	**0.0003**	0.0012	0.0166

Significant values are in bold.

### Graphical illustration and discussion

In this part, we present the numerical results derived in above example graphically in order to verify the effectiveness and essential differences among them.

Figures 1, 2 and 3 also indicate various significant results. When employing different distance measures, one can notice that the calculated values are increasing or decreasing as the value of $\lambda$ fluctuates. For instance, if we employ the generalized weighted distance measure to determine the distances (shown as Fig. 1), the calculated values are monotonically decreasing as the parameter $\lambda$ increases and vice versa. Analogous results can be obtained from Figs. 2, and 3 also. Therefore, from this aspect, the parameter $\lambda$ can be deemed as a DM’s risk attitude. Thus, the presented distance measures give the DMs more choices as they can choose the value of $\lambda$ according to their preferences.

It is mandatory for us to compare all the distance and similarity measures studied in this article for ease of implementation. All the measures proposed in section “Distance and similarity measures between two NWDHFSs” are basically about the distance and similarity measures between two NWHFSs. The measures discussed in section “Weighted distance and similarity measures between two NWDHFSs” have a weighted form as there are different types of NWDHFEs in each set $N_{r} (r=1,2)$, and the NWDHFEs within the set may have various importance grades. The normal wiggly dual hesitant Hamming distance and the normal wiggly dual hesitant Euclidean distance are the specific cases of the generalized normal wiggly dual hesitant distance measure, using $\lambda =1$ and $\lambda =2$, respectively. The normal wiggly dual hesitant Hamming Hausdorff distance and the normal wiggly dual hesitant Euclidean Hausdorff distance are also the particular cases of the generalized normal wiggly dual hesitant Hausdorff distance measure in the sense that taking $\lambda =1$ and $\lambda =2$, respectively. In the same manner, the hybrid normal wiggly dual hesitant Hamming distance and the hybrid normal wiggly dual hesitant Hausdorff distance are also specific cases of the generalized hybrid normal wiggly dual hesitant distance measure with $\lambda =1$ and $\lambda =2$, respectively. The Hausdorff distance is known as the largest distance. If we pay attention to the values in the three Tables 6, 7 and 8, it is easy to figure out that the generalized normal wiggly dual hesitant Hausdorff weighted distances are much bigger than the corresponding generalized normal wiggly dual hesitant weighted and generalized Hybrid normal wiggly dual hesitant weighted distances. The distance measures introduced in section “Weighted distance and similarity measures between two NWDHFSs in continuous case” also have the same properties, i.e., the generalized normal wiggly dual hesitant weighted distance, generalized normal wiggly dual hesitant weighted Hausdorff distance, and the generalized hybrid normal wiggly dual hesitant weighted distance are the fundamental types of the weighted distance measure between NWDHFSs, and the rest are their particular cases. When the weights are provided in discrete form, one can employ the measures presented in chapter 4.1, while if the weights are given in the continuous form, then the continuous distance measure studied in section “Weighted distance and similarity measures between two NWDHFSs in continuous case” can be used.

### Comparison

In order to analyze the ranking results thoroughly and illustrate the advantages of the proposed distance measures, this part compares the above decision-making results with the results produced by the classical distance measures^60,65^.

We first employ the Wang et al.^60^, distance measures to solve the problem given in section “Practical example”. The decision matrices Tables 2 and 3, and the weight vector $w=(0.3,0.3,0.2,0.2)^T$ are used to derive diagnosis for suspected patients. The generalized dual hesitant distance, generalized dual hesitant distances with preference and weighted generalized dual hesitant distances with preference $(\alpha =\beta =1/2)$^60^ are used to seek diagnoses for the three patients according to various values of $\lambda$. The obtained results are depicted in Tables 9, 10, and 11.

**Table 9:** The generalized dual hesitant distances among patients and diseases.

	$\lambda =1$			$\lambda =2$			$\lambda =4$			$\lambda =6$		
	$P_1$	$P_2$	$P_3$	$P_1$	$P_2$	$P_3$	$P_1$	$P_2$	$P_3$	$P_1$	$P_2$	$P_3$
$D_1$	0.2074	0.1524	**0.1072**	0.2648	0.2073	**0.1567**	0.3099	0.2659	**0.2078**	0.3318	0.3012	**0.2349**
$D_2$	0.1632	**0.1312**	0.1536	0.2160	**0.1742**	0.2088	0.2613	**0.2199**	0.2605	0.2815	**0.2453**	0.2864
$D_3$	**0.1311**	0.1754	0.2354	**0.1893**	0.2187	0.2932	**0.2396**	0.2563	0.3508	**0.2627**	0.2750	0.3864

Significant values are in bold.

**Table 10:** The generalized dual hesitant distances with preference among patients and diseases.

	$\lambda =1$			$\lambda =2$			$\lambda =4$			$\lambda =6$		
	$P_1$	$P_2$	$P_3$	$P_1$	$P_2$	$P_3$	$P_1$	$P_2$	$P_3$	$P_1$	$P_2$	$P_3$
$D_1$	0.1037	0.0762	**0.0536**	0.1872	0.1466	**0.1108**	0.2606	0.2236	**0.1747**	0.2956	0.2683	**0.2093**
$D_2$	0.0816	**0.0656**	0.0768	0.1527	**0.1232**	0.1477	0.2197	**0.1849**	0.2191	0.2508	**0.2185**	0.2552
$D_3$	**0.0655**	0.0877	0.1177	**0.1339**	0.1546	0.2073	**0.2015**	0.2155	0.2949	**0.2341**	0.2450	0.3443

Significant values are in bold.

**Table 11:** The weighted generalized dual hesitant distances with preference among patients and diseases.

	$\lambda =1$			$\lambda =2$			$\lambda =4$			$\lambda =6$		
	$P_1$	$P_2$	$P_3$	$P_1$	$P_2$	$P_3$	$P_1$	$P_2$	$P_3$	$P_1$	$P_2$	$P_3$
$D_1$	0.0244	0.0192	**0.0147**	0.0899	0.0728	**0.0591**	0.1795	0.1553	**0.1286**	0.2296	0.2086	**0.1710**
$D_2$	0.0201	**0.0158**	0.0178	0.0761	**0.0598**	0.0697	0.1561	**0.1285**	0.1493	0.2003	**0.1714**	0.1972
$D_3$	**0.0172**	0.0877	0.0288	**0.0691**	0.0752	0.1009	**0.1454**	0.1496	0.2027	**0.1887**	0.1917	0.2659

Significant values are in bold.

It is easy to see from Tables 9, 10 and 11 that the results derived by Wang et al.’s measures are totally the same as normal wiggly dual hesitant fuzzy measures. This discloses that the proposed distance measures are appropriate and practicable. Likewise, the proposed measures, Wang et al.’s measures are also stable with a variation in $\lambda$, as can be seen from Tables 9, 10, and 11. Though the results are exactly the same, the discrimination of Wang et al.’s measures is evidently weaker than our designed measures. The cause of this is that Wang et al.’s measures are based only on dual hesitant fuzzy information, while the proposed measures are constructed for normal wiggly dual hesitant fuzzy data. Thus, in this case, DMs’ deeper uncertain information coupled with DHFSs is necessary to achieve more rational outcomes. The normal wiggly dual hesitant fuzzy information covers the dual hesitant fuzzy information and consists of uncertain information, which is revealed using rational methods from the original dual hesitant fuzzy information. The distance measures^60^ used in Table 10 and 11 have an extra advantage of the preference coefficients, which can be decided accordant with DMs’ psychological preference, but these coefficients are users defined values, and the results are also sensitive to these values (can be seen in^60^). Thereby, DMs need some training before using these measures because the authors have not provided specific guidelines to the users about selecting these coefficients in the manuscript.

To further compare the performance of the proposed measures, we utilize the distance measures outlined by^65^ to address the provided problem.

Since Yang et al.^65^ measures consider only the membership parts of NWDHFSs, therefore, we take advantage of only normal wiggly hesitant fuzzy data of Tables 4 and 5 while deriving the desired results. Further, the weights of criteria are taken as that considered in the proposed distance measures.

**Table 12:** The generalized normal wiggly hesitant distances with preference among patients and diseases.

	$\lambda =1$			$\lambda =2$			$\lambda =4$			$\lambda =6$		
	$P_1$	$P_2$	$P_3$	$P_1$	$P_2$	$P_3$	$P_1$	$P_2$	$P_3$	$P_1$	$P_2$	$P_3$
$D_1$	0.2889	0.3155	**0.2817**	0.3225	0.3065	**0.2892**	0.3640	0.3135	**0.3030**	0.4080	0.3215	**0.3148**
$D_2$	0.1710	**0.1633**	0.3277	0.1658	**0.1659**	0.3064	**0.1735**	**0.1921**	0.3134	**0.1800**	**0.2151**	0.3243
$D_3$	**0.1431**	0.2209	0.3483	**0.1547**	0.2101	0.3876	0.1940	0.2249	0.4398	0.2260	0.2404	0.4722

Significant values are in bold.

**Table 13:** The weighted generalized normal wiggly hesitant distances with preference among patients and diseases.

	$\lambda =1$			$\lambda =2$			$\lambda =4$			$\lambda =6$		
	$P_1$	$P_2$	$P_3$	$P_1$	$P_2$	$P_3$	$P_1$	$P_2$	$P_3$	$P_1$	$P_2$	$P_3$
$D_1$	0.1853	0.2291	**0.2286**	0.2535	0.2628	**0.2623**	0.3209	0.2932	**0.2882**	0.3748	0.3095	**0.3045**
$D_2$	0.1240	**0.1265**	0.2451	**0.1407**	**0.1475**	0.2636	**0.1596**	**0.1834**	0.2903	**0.1702**	**0.2099**	0.3069
$D_3$	**0.1134**	0.1640	0.2587	0.1415	0.1794	0.3238	0.1883	0.2059	0.3941	0.2221	0.2258	0.4360

Significant values are in bold.

The obtained results are depicted in Tables 12 and 13. Evidently, the results derived by Yang et al. ’s measures are roughly the same as derived by the proposed measures, which validates the efficiency of devised measures. However, from Table 12, we find that the patient $p_1$ suffers from disease $D_2$ instead of disease $D_1$ by taking the parameter values $\lambda =4,6$. Apart from this, similar situation occurs in Table 13 also for $\lambda =2,4,6$. Thus, by varying the values of $\lambda$, the results derived by^65^ measures are unstable. This is because these measures only consider the membership part and its corresponding deeper uncertain information but ignore the non-membership aspect, which loss the original information to a certain degree. On the other hand, the proposed measures are based on normal wiggly dual hesitant fuzzy setting, which reserves all the dual hesitant fuzzy information. However, as discussed before, the existing measures^60,65^ have an extra advantage of the DMs’ psychological preferences factor, which plays a key role while making decisions.

From the above comparative study, the designed measures have the following advantages:

1. Potential information: The proposed measures context not only allows DMs to assign assessment values like DHFS but also reflects the potential information hidden behind the original data, which facilitates them to obtain a more accurate result.
2. Stability: According to Tables 6, 7, and 8 the computed distance measures between the patients and the diseases decreases with the increasing value of $\lambda$, but the diagnosis remains unaltered. This phenomenon demonstrates the stability and rationality of the designed measures.
3. Rapidity: The presented measures take less time in the data-processing technique and better model emergency decision-making problems.

The provided measures also suffer from certain disadvantages as listed below:

1. Artificial values: In the proposed measures, shorter DHFEs are extended to the same length by including some artificial values. The inclusion of these artificial values increases the evaluation process’s subjectivity and may yield irrational results.
2. DMs’ psychological preferences: The presented mathematical formulations ignore the DMs’ psychological preferences factor^60,65^, which plays a key role while making decisions.

## Concluding remarks and suggestions

NWDHFS, as an extension of DHFS, can not only retains the original dual hesitant fuzzy information but also obtains valuable uncertain information, which can help DMs to express the evaluation information more completely. The present work introduced several distance and similarity measures under the background of NWDHFS. After elaborating the refined operational laws, we first generalized the extension rule of length for normal wiggly dual hesitant fuzzy setting and then studied a series of distance measures for NWDHFSs based on the ideas of the well-known Hamming distance, the Euclidean distance, the Hausdorff metric, the hybrid distance, and their extensions. Subsequently, we proposed a family of weighted version distance measures for both discrete and continuous cases and discussed their special cases in depth. It should be noted that we have concentrated our attention on distance measures in this study, while the corresponding similarity measures for NWDHFSs can be obtained through the relationship between the distance measures and similarity measures. In the end, a practical example of medical diagnosis is addressed to show the validity and practicality of the developed distance and similarity measures with a detailed discussion of the parameter influence. From the numerical results, we observed that the parameter $\lambda$ can be considered as a DM’s risk attitude. Consequently, the proposed distance measures offer more choices to DMs as the parameter regarding DM’s risk preferences is provided.

The following lines may be the directions for future research.

1. Because of the importance of hesitance degree^66^, one can introduce the concept of hesitance degree of NWDHFS and can propose several novel distance and similarity measures between NWDHFSs by including the hesitance degree.
2. Likewise the hesitance degree, researchers can also investigate distance and similarity measures in terms of preference coefficients to tackle the situation where DMs have different preferences between the hesitant part and wiggly part^28^.
3. By extending the matching function^67^ to NWDHFS context, experts can also study the similarity measure for NWDHFSs based on the matching function. Further, the research on the theory of credibility degree^68^ is also inevitable.
4. The construction of ordered weighted distance and similarity measures^69^ under the background of NWDHFS is an open problem.
5. The method and principle of finding the DM’s weights, including the numerical effects of the weights and how to assign appropriate weights for different DMs, may also be the direction for future research.

## Data Availability

All data generated or analyzed during this study are included in this published article.
